# Over forty years of bladder cancer glycobiology: Where do glycans stand facing precision oncology?

**DOI:** 10.18632/oncotarget.19433

**Published:** 2017-07-21

**Authors:** Rita Azevedo, Andreia Peixoto, Cristiana Gaiteiro, Elisabete Fernandes, Manuel Neves, Luís Lima, Lúcio Lara Santos, José Alexandre Ferreira

**Affiliations:** ^1^ Experimental Pathology and Therapeutics Group, Portuguese Institute of Oncology, Porto, Portugal; ^2^ Institute of Biomedical Sciences Abel Salazar, University of Porto, Porto, Portugal; ^3^ New Therapies Group, INEB-Institute for Biomedical Engineering, Porto, Portugal; ^4^ Instituto de Investigação e Inovação em Saúde, Universidade do Porto, Porto, Portugal; ^5^ Biomaterials for Multistage Drug and Cell Delivery, INEB-Institute for Biomedical Engineering, Porto, Portugal; ^6^ Glycobiology in Cancer, Institute of Molecular Pathology and Immunology of the University of Porto, Porto, Portugal; ^7^ Department of Surgical Oncology, Portuguese Institute of Oncology, Porto, Portugal

**Keywords:** cancer glycobiology, bladder cancer, glycoproteomics, glycomics, precision medicine

## Abstract

The high molecular heterogeneity of bladder tumours is responsible for significant variations in disease course, as well as elevated recurrence and progression rates, thereby hampering the introduction of more effective targeted therapeutics. The implementation of precision oncology settings supported by robust molecular models for individualization of patient management is warranted. This effort requires a comprehensive integration of large sets of panomics data that is yet to be fully achieved. Contributing to this goal, over 40 years of bladder cancer glycobiology have disclosed a plethora of cancer-specific glycans and glycoconjugates (glycoproteins, glycolipids, proteoglycans) accompanying disease progressions and dissemination. This review comprehensively addresses the main structural findings in the field and consequent biological and clinical implications. Given the cell surface and secreted nature of these molecules, we further discuss their potential for non-invasive detection and therapeutic development. Moreover, we highlight novel mass-spectrometry-based high-throughput analytical and bioinformatics tools to interrogate the glycome in the postgenomic era. Ultimately, we outline a roadmap to guide future developments in glycomics envisaging clinical implementation.

## INTRODUCTION

**Figure 1:**
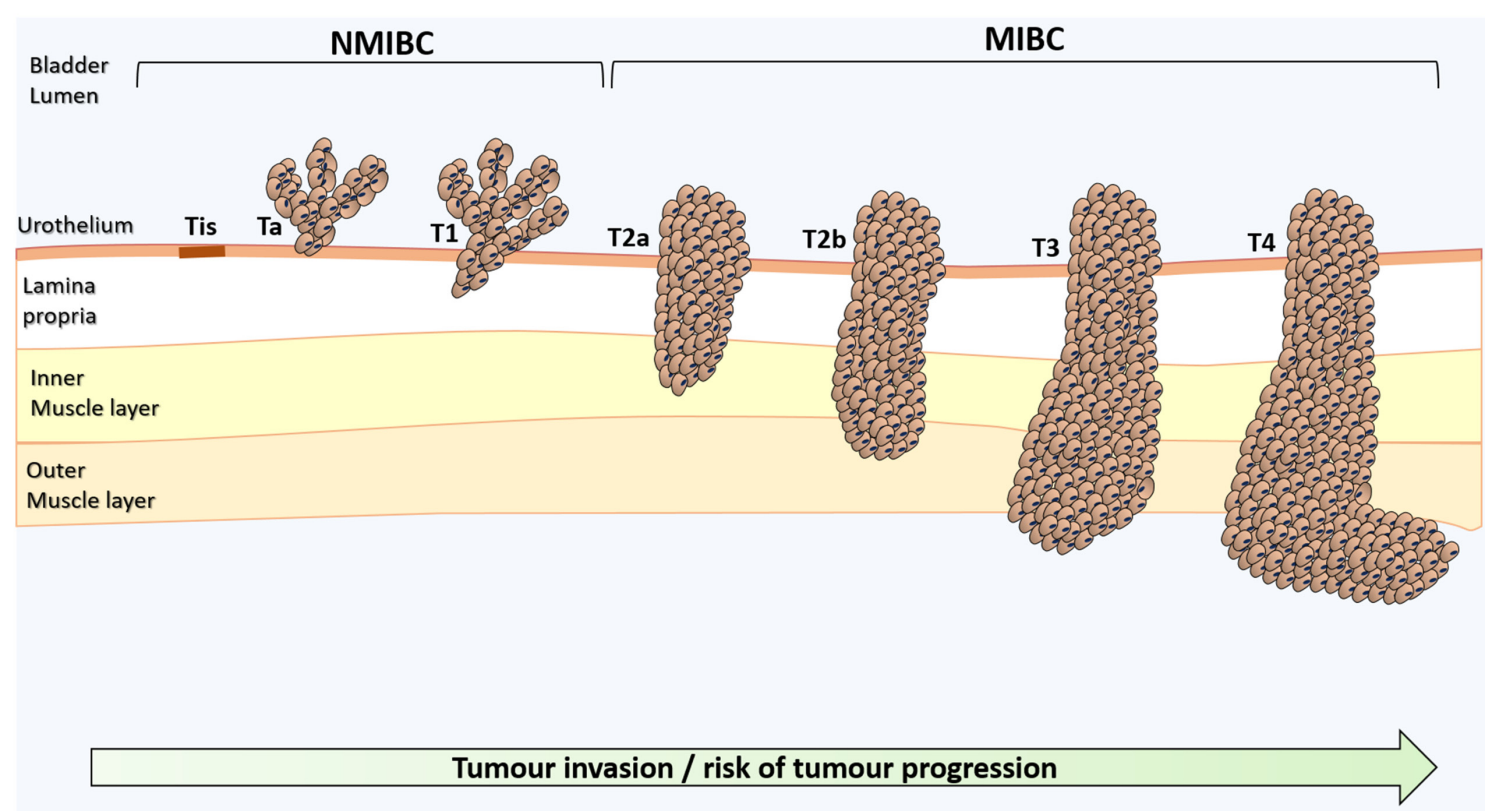
Schematic representation of bladder cancer stage and grade The stage of the primary tumour (T) is based on the extent of penetration or invasion into the bladder wall. Regarding tumour grading, bladder lesions can be classified as urothelial papilloma (a benign lesion), papillary urothelial neoplasm of low malignant potential (PUNLMP), low-grade papillary urothelial carcinoma and high-grade papillary urothelial carcinoma. Of note, PUNLMP lesions do not have cytological features of malignancy and have a very low risk of progression. Nevertheless, they show high tendency to recur. Tis, Tumour in situ: ‘‘flat tumour’’; Ta, Non-invasive papillary carcinoma; T1, Tumour invades sub-epithelial connective tissue; T2, Tumour invades muscle; T2a, Tumour invades superficial muscle (inner half); T2b, Tumour invades deep muscle (outer half); T3, Tumour invades perivesical tissue; T4, Tumour invades any of the following: prostate, uterus, vagina, pelvic or abdominal wall.

Bladder cancer, particularly muscle invasive bladder cancer (MIBC), is amongst the most common and deadliest genitourinary cancers [1]. The mainstay treatment for advanced stage tumours includes surgery and cisplatin-based chemotherapeutic regimens [1], which fail in avoiding tumour relapse and disease progression. Tremendous efforts have been put in the establishment of biomarker panels for early diagnosis, follow-up, patient stratification, prognosis, treatment selection and development of targeted therapeutics [2]. However, the highly heterogeneous molecular nature of bladder tumours has hampered true developments in this field [3]. Moreover, bladder cancer remains mostly an “orphan disease” in terms of targeted therapeutics, leading to few improvements in patient’s overall survival over the last decade [2, 4]. More detailed information on the clinicopathological nature of bladder tumours and critical aspects in disease management have been recently reviewed [5]. A schematic illustration of bladder cancer staging and grading is shown in Figure 1.

Several decades of glycobiology research have disclosed the existence of profound alterations in the glycosylation patterns of bladder tumours, reflecting specific changes in glycan biosynthetic pathways, glycosyltransferases expression, amongst other factors [6]. These events often lead to novel protein and lipid glycoforms, either by incomplete or neo-synthesis of glycan epitopes, that cannot be found in the corresponding healthy tissues and preneoplastic lesions. These events play a key role in tumour progression by affecting ligand-receptor interactions, and interfering with regulation of cell signaling, adhesion, migration, proliferation, angiogenesis, and immune responses [6]. Moreover, cancer-associated glycans may be actively secreted into bodily fluids (e.g. blood and urine) or shed from apoptotic and necrotic cancer cells [7]. As such, glycans and abnormally glycosylated molecules (e.g. proteins and lipids) hold tremendous value for non-invasive cancer detection, while membrane bound glycans may be used to selectively target tumour sites and specific cancer cells. Nevertheless, the structural complexity and heterogeneity of oligosaccharides, and the lack of analytical methods for elucidating structures still pose a major difficulty when addressing the glycome, glycolipidome and glycoproteome [8]. Still, a plethora of mass spectrometry-based analytical approaches have been developed to address these challenges [8, 9] and the standardization of high-throughput glycomics is expected to boost our knowledge on bladder cancer glycobiology in the near future.

Based on these considerations, the present review comprehensively summarizes the clinical significance of the main biomarkers arising from over forty years of bladder cancer glycobiology research and establishes the milestones towards clinical applications. Ultimately, we discuss the need to integrate glycans in holistic panomics models for precision oncology, namely the molecular-based individualization of patient care.

### Glycosylation signatures in bladder cancer: biological and clinical implications

Glycosylation is the most frequent, complex and plastic post-translational modification of secreted and membrane-bound proteins, as well as a common substitution in lipids at the cell membrane [10]. Glycans are secondary gene products resulting from the coordinated action of nucleotide sugar transporters, glycosyltransferases and glycosidases in the endoplasmic reticulum (ER) and Golgi apparatus (GA) of mammalian cells [10]. Glycans are involved in several structural, modulatory, molecular mimicry and recognition roles including protein folding, stability, adhesion and trafficking, as recently reviewed [11]. Alterations in glycosylation patterns are common features of solid tumours, being detected even in pre-malignant lesions [12]. Generally, the most frequently described cancer-related glycosylation modifications include the synthesis of highly branched and heavily sialylated glycans, the premature termination of biosynthesis, resulting in the expression of short-chained forms, and the expression *de-novo* of glycosidic antigens of foetal type [13]. These structural motifs are mostly associated with: i) altered glycogenes expression [14, 15]; ii) impaired glycosyltransferases’ chaperone function [16]; iii) altered glycosidase/glycosyltransferase activity [15]; iv) reorganization of glycosyltransferases topology [17, 18]; v) bioavailability of sugar nucleotide donors and cofactors [19]; vi) alterations on the conformation of peptide backbone or on the nascent glycan chain structure [19]. The resultant aberrant and cancer-associated glycans seem to be implicated in the activation of oncogenic pathways [20], establishment of tumour-tolerogenic immune responses [21], and in epithelial-to-mesenchymal transition (EMT), a crucial milestone towards invasion and metastasis [22, 23]. Thus, many glycoepitopes, and their related glycosidases/glycosyltransferases, can be considered relevant tumour-associated antigens [24, 25], with possible clinical significance in bladder cancer. Therefore, the following sections will focus on these key findings in bladder cancer glycobiology (summarized in Supplementary Table 1 - Supplementary material). Given their structural complexity and broad distribution, known cancer-associated glycogenes, glycosyltransferases and glycans will be presented in the context of specific classes of biomolecules (glycoproteins, glycolipids, proteoglycans).

### Protein glycosylation

Two main classes of glycans can be found altered in cancer cell-surface proteins, namely *N*-glycans, attached to the peptide sequence via an asparagine (Asn) residue, and *O*-glycans, attached by a *N*-acetylgalactosamine (GalNAc) residue to the hydroxyl group of a serine (Ser) or threonine (Thr) residue.

#### Cancer-associated N-glycans

**Figure 2:**
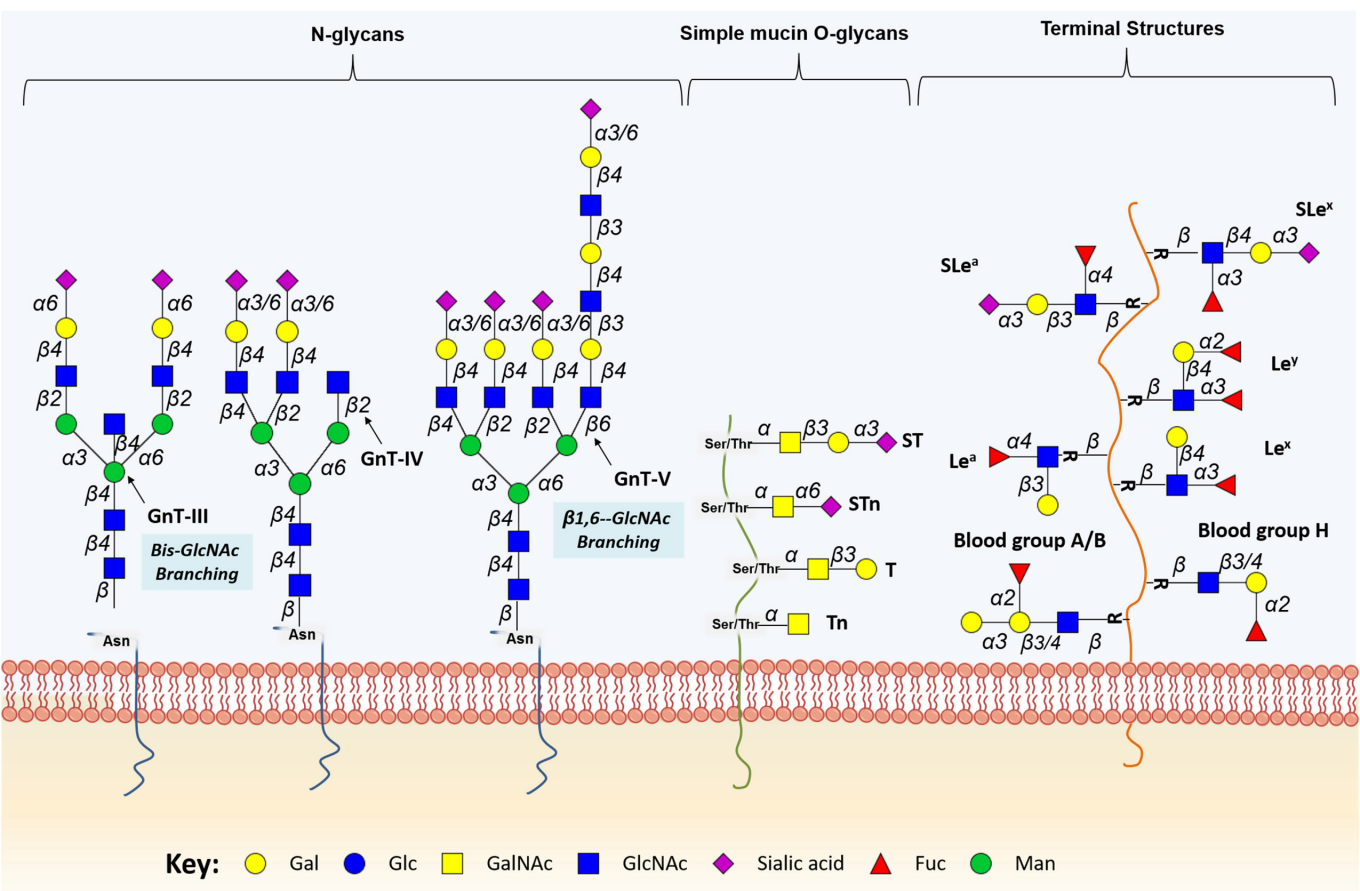
Schematic representation of protein-associated glycan structures relevant in bladder cancer The figure represents specific *N*-linked and *O*-linked glycan structures, as well as terminal Lewis and sialylated Lewis structures that have biological significance in bladder cancer. Key enzymes mediating the addition of specific sugars are also shown. Protein *N*-glycan alterations include the β1-6 branching of *N*-glycans in result of GlcNAcT-V (GnT-V) overexpression, and the addition of bisecting GlcNAc branches by GlcNAcT-III (GnT-III) glycosyltransferases. Alterations in O-glycosylation pathways are also a common hallmark of malignant transformations of the bladder. Herein, we represent the overexpression of simple mucin-type O-glycans and their sialylated counterparts, T, sialyl T (ST), Tn and sialyl Tn (STn) antigens. Altered expression of terminal structures is also a common feature of bladder tumours. Namely, the abnormally low or absent expression of ABO(H) blood group determinants is frequently found in high grade and invasive disease. Carbohydrate terminal Lewis antigens are significantly under-expressed in healthy urothelium when compared to bladder tumours and are also highlighted here. Lewis type 1 antigens include Lewis^a^ (Le^a^), and sialyl Lewis^a^ (SLe^a^), while the type 2 group includes Lewis^x^ (Le^x^) and sialyl Lewis^x^ (Sle^x^).

Protein *N*-glycosylation takes place in the ER, where the oligosaccharide transferase complex (OSTase) scans nascent proteins for Asn-X-Ser/Thr “sequons” (“X” stands for any amino acid residue except proline), and transfers a precursor glycan (Glc_3_Man_9_GlcNAc_2_-) from dolichol pyrophosphate to Asn residues [26]. At this point, all *N*-glycans share a common core structure (Manα1-6(Manα1-3)Manβ1-4GlcNAcβ1-4GlcNAcβ1-Asn-X-Ser/Thr), which is further processed in the ER and GA by several glycosyltransferases and glycosidases, yielding mature core structures that may be classified into three major *N*-glycan types (oligomannose, complex, and hybrid, Figure 2). The *O*-3 linked Man residues in hybrid and complex *N*-glycans may be further *O*-4 substituted with *N*-acetylglucosamine (GlcNAc) residues by GlcNAcT-III (GnT-III) to yield bisecting core structures. The introduction of the bisecting GlcNAc residue by GnT-III alters the composition and conformation of the *N*-glycan, resulting in the suppression of further processing and elongation [27, 28]. More highly branched *N*-glycans may be generated by the action of different GlcNActransferases (GnT-IV, -V, -VI). These structures may be further elongated with galactose, poly-*N*-acetyllactosamine, sialic acid, and fucose residues. Particularly, *N*-glycans frequently exhibit Lewis (Le) blood group related antigens (Le^a^, Le^x^, Le^b^ and Le^y^) and corresponding sialylated structures or ABO(H) blood group determinants as terminal epitopes. Similar terminal structures may also be found in *O*-glycans (Figure 2). Other sugar modifications may include phosphorylation, *O*-acetylation of sialic acids, and *O*-sulfation of galactose and *N*-acetylglucosamine residues, thereby increasing the structural complexity of the glycome [29].

Several *N*-glycan alterations have been described in bladder tumours, including changes in branching and terminal structures through oversialylation, fucosylation (Supplementary Table 1), which will be discussed in detail in the following sections.

#### *N*-glycans branching

Alterations in *N*-glycans branching resulting from impaired GnTs expression have been evaluated in the context of bladder cancer prognosis. Namely, increased GnT-III, *N*-glycans bisection and GnT-IV expression were associated with higher disease stage and grade in bladder cancer patients [30]. Conversely, decreased GnT-V expression, responsible by *O*-6 *N*-glycans branching, was found associated with higher bladder tumour grade and stage, shorter disease-free survival and bladder cancer recurrence [31, 32]. Moreover, low GnT-V expression was found to predict shorter cause-specific survival of bladder cancer patients while overexpression of *O*-6 branched *N*-linked oligosaccharides was associated with lower tumour stage, suggesting that these findings could be applied to risk stratification [32]. The opposing associations of GnT-III and GnT-V in bladder cancer prognosis can be explained by the antagonistic effect of their enzymatic activity [28]. Contrasting with the findings for bladder cancer, reduced GnT-III and increased GnT-V expressions have been found to promote metastasis in different cancer models [33-36] yet no consensus exists between GnT-V expression and prognosis in gastric [35, 36], oral squamous cell [37] and endometrial cancers [38]. These observations suggest GnT-V/III evaluation may hold potential for bladder cancer prognosis and ultimately targeted therapeutics, which warrants confirmation in future studies.

#### b) *N*-glycans terminal structures (also found in protein *O*-glycans and glycolipids)

Amongst the most common cancer-associated structural features are alterations of terminal glycan epitopes. In fact, the first reported glycosylation alterations in bladder cancer were the loss of ABO(H) blood group determinants in advanced stage carcinomas of secretor individuals [39, 40], as well as changes in Lewis antigens patterns.

The ABO(H) blood group system consists of terminal oligosaccharide antigens carried by glycoproteins or glycolipids in hematopoietic or epithelial cells [41]. Their biosynthesis is presumed to be controlled by the *ABO(H), Se, H, Le*, and *X* blood group genes [41]. These antigens are present on normal bladder epithelium of secretor individuals but not on some low-grade and early-stage papillary urothelial carcinomas [42]. Moreover, initially expressing tumours lose these cell surface antigens upon local recurrence, progression to invasion or metastization [42]. As such, the possibility that loss of genetically predicted blood group antigens precedes the development of recurrent, invasive or metastatic bladder cancer has been extensively explored [43]. Studies have shown that abnormally low or absent expression of these epitopes is frequently found in high grade and invasive bladder disease [44-46] and associated with bladder tumour progression and shorter recurrence-free survival [47]. Furthermore, loss of tissue ABO(H) antigens in the initial biopsy of bladder carcinomas predicts a much greater chance of subsequent invasion than in tumours with detectable ABO(H) antigens [44, 45, 47]. However, a significant number of patients whose initial tumours were reported as blood group antigen negative failed to develop an invasive tumour [47]. It is possible that these conflicting results may, at least in part, be explained by differences in methodology, interpretation, or both. Moreover, the loss of activity of the *A* and *B* gene-encoded transferases in bladder tumours from blood group A and B individuals was reported, which explains the deletion of these antigens in bladder tumours [48]. In addition, the loss of the ABO(H) gene and/or its promoter hypermethylation is a specific marker for urothelial carcinoma [39]. In summary, alterations in ABO(H) accompanying bladder malignant transformation and disease dissemination are well established surrogate markers of profound alterations in glycosylation pathways, constituting important starting points for more in depth structural studies.

The ABO(H) determinants have biosynthetic and structural similarities with Lewis antigens, including the fucosylated type 1 Lewis^a^ (Galβ(1-3)GlcNAc[Fucα(1-4)]) and type 2 Lewis^x^ (Galβ(1-3)GlcNAc[Fucα(1-4)]). Several authors have associated Lewis^a^ and Lewis^x^ expression patterns with malignant transformations of the bladder, reporting significantly lower expression of this antigen in healthy urothelium when compared to invasive tumours [44, 46]. As such, reduced expression of Lewis^a^ and Lewis^x^ was associated with higher tumour grade and invasion [44] and shorter recurrence-free survival [49]. As such, the expression of these antigens can be associated with worse bladder cancer phenotypes. Moreover, Lewis^a^ antigen expression patterns change at an early neoplastic stage, suggesting that Lewis^a^ determination might be useful in the diagnosis of very early premalignant changes in the urothelium [49]. In addition, scoring Lewis^a^ expression allows the sub-classification of histologically identical tumours into prognostically different groups, pointing to a relationship between the pathological grade and stage of the evaluated tumours and a morphological and functional de-differentiation [49]. Given this, Lewis^a^ antigen is a valuable functional marker of the malignant potential in superficial bladder cancer. In turn, the Lewis^x^ antigen is not expressed in normal urothelium, except for occasional umbrella cells [46, 50], but has been found in the majority of invasive tumours, regardless of blood type and secretor status of the individuals studied [46]. Lewis^y^ is expressed in both normal urothelium and bladder tumours, yet its expression was associated with bladder tumour invasion capability [46]. Nevertheless, the number of studies concerning Lewis antigens in bladder cancer is still scarce to withdraw conclusions about their biological and clinical significance.

#### c) Oversialylation and fucosylation (also occurring in protein *O*-glycans and glycolipids)

Oversialylation of cancer cells often stem from the overexpression of sialylated Lewis antigens sialyl lewis^a^ (SLe^a^; the CA19-9 antigen) and sialyl lewis^x^ (SLe^x^), which can be found as terminal epitopes of *N*-glycans, *O*-glycans and glycolipids [51]. SLe^a/x^ are specific ligands for E- and P-selectins in endothelial cells, thereby promoting the adhesion of malignant cells to the endothelium and the metastatic cascade [50-52]. These antigens also thought to play a role in tumour growth, invasion, and angiogenesis [51, 53]. In line with these observations, the overexpression of SLe^a^ and SLe^x^ have also been associated with bladder cancer malignant potential. Particularly, serum overexpression of SLe^a^ was associated with higher stage, grade and invasion [53] while tissue loss/reduction of SLe^a^ expression was associated with higher atypia grade [50]. SLe^x^ has been closely link to invasive and metastatic potential of primary bladder tumours and correlated with shorter 5-year and 7-year survival rates [54], but another study demonstrated no associations between SLe^x^ with grade or stage in urothelial carcinoma of the renal pelvis, ureter, and urinary bladder [50]. The disialylated form of Le^a^ (termed disialyl-Lewis^a^, dSLe^a^) was described as preferentially expressed in non-malignant cells, and may be useful for distinguishing benign from malignant diseases mostly expressing SLe^a^ [55]. Supporting these observations, the overall increase in cell-surface sialic acid content was shown to reduce the attachment of metastatic tumour cells to the extracellular matrix [56]. These observations support the need for a comprehensive interrogation of bladder cancer cells “sialome” towards understanding tumour progression and dissemination. Moreover, future studies should explore the biological and clinical relevance of structurally identical sialylated forms in the context of bladder cancer.

Fucosylation is another common modification involving oligosaccharides on glycoproteins and glycolipids [57]. Particularly, the quantitative glycome analysis of *N*-glycan patterns in bladder cancer cells often reveals significant differences in *N*-glycan fucosylation compared to normal cells. Namely, bladder cancer cells (KK47, YTS1, J82, T24) showed high expression of complex core-fucosylated *N*-glycans and low expression of terminally fucosylated *N*-glycans [58]. Nevertheless, the implications of these differential fucosylation patterns in bladder cancer malignancy have been so far poorly explored. The transcript levels of fucosyltransferase (FUT) VI (*FUT-VI*) and *FUT-VII* from invasive and non-invasive bladder tumours were also explored using RT-PCR. Particularly, bladder cancer cell lines from invasive tumours that maintained their metastatic properties showed high levels of both enzymes, and cell lines from non-invasive tumours (KK-47) or normal bladder epithelia (HCV-29) were negative for *FUT-VI* and *FUT-VII* [54]. These evidences suggest that *FUT-VI/-VII* expression associates with more malignant cancer cell phenotypes. Another study has described β1-integrin activation by alpha1,2-fucosyltransferase 1 (FUT-I)-mediated fucosylation in J82 human bladder cancer cells, thereby enhancing bladder cancer adhesion and subsequent metastasis [59]. As such, changes in bladder cancer fucosylation patterns seem to be associated with tumour invasion and progression to metastization in cancer cell lines, suggesting that these changes could provide novel strategies for cancer therapy.

#### Cancer-associated *O*-glycosylation

**Figure 3:**
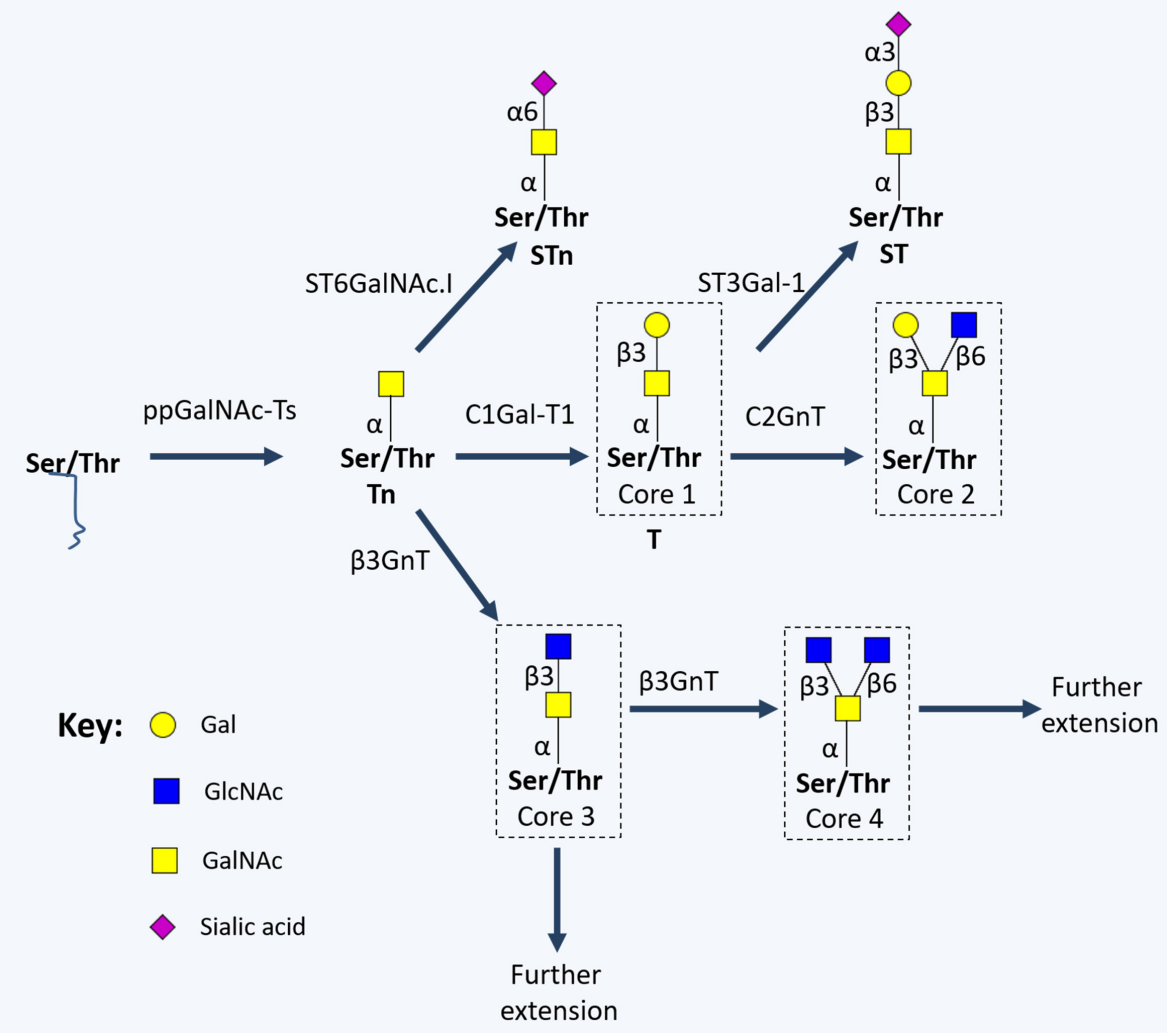
Schematic representation of short-chained O-linked glycan structures The addition of specific sugar monomers to Ser/Thr residues of a protein backbone begins with the action of polypeptide N-acetylgalactosamine transferases (ppGalNAc-Ts; a family of 20 enzymes, including GalNAc-T1, GalNAc-T2, GalNAc-T3, GalNAc-T4, GalNAc-T5 and GalNAc-T6) giving rise to the Tn antigen, which is generally extended with a Gal residue by C1Gal-T1, originating the Thomsen-Friedenreich or T antigen (core 1). Alternatively, Tn and T antigens can be sialylated by α2,3-sialyltransferases (ST3Gal-Ts) and α-GalNAc ST6Gal-I (ST6GalNAc-I), forming the sialyl-Tn (STn), and sialyl-T antigens. On the other hand, core 1 may be extended originating cores 2-4 by the action of N-acetylglucosamine (GlcNAc) transferases (GnTs; such as GnT-III, GnT-V, core 2 GnTs (C2GnTs) and β3GnT).

The most common form of cell-surface protein *O*-glycosylation results from the transfer of a GalNAc residue from a UDP-GalNAc donor to either serine or threonine in a given polypeptide chain (*O*-GalNAc glycosylation), originating the monosaccharide Tn antigen. This reaction is catalysed by several UDP-GalNAc:polypeptide *N*-acetylgalactosaminyl transferases (ppGalNAc-Ts) in the ER, in a substrate dependent manner [60]. As opposed to *N*-glycosylation, no consensus sequence is required for ppGalNAc-Ts recognition. The Tn antigen is generally extended with a Gal residue by Gal-transferase (β(1-3)-galactosyltransferase, C1Gal-T1 or T-synthase) and cosmc chaperone, originating the disaccharide Thomsen-Friedenreich or T antigen (Galβ1-3GalNAcα-O-Ser/Thr, core 1). Alternatively, Tn and T antigens can be sialylated by sialyltransferases, forming the sialyl-Tn (STn), sialyl-T and disialyl-T antigens. Sialylation stops any further processing of the oligosaccharide chain, prompting short-chain GalNAc-type *O*-glycans expression [60]. Alternatively, core 1 may be extended originating cores 2-4 (Figure 3), which are precursors for a vast array of more extended oligosaccharides and terminal structures, similar to the ones found in mature N-glycans.

Recently, a precision mapping of human *O*-GalNAc glycoproteome has revealed over 6000 glycosites in more than 600 *O*-glycoproteins, the majority of which of membrane origin [61], greatly expanding our view on the *O*-glycoproteome and its functional role. Alterations in *O*-glycosylation pathways are a common hallmark of malignant transformations, frequently amplified at the cell-surface as a result of the high number of *O*-glycosylation sites presented by mucins [62]. Such events are particularly pronounced in adenocarcinomas, due to the overexpression of these molecules [63]. While hindered by extended glycosylation in healthy and benign tissues, simple mucin-type *O*-GalNAc glycans are uncovered in most human carcinomas, including bladder cancer [45, 64-67].

#### a) Premature stop in *O*-glycosylation

Perhaps the most studied cancer-associated *O*-glycans are the Tn antigen, its sialylated counterpart sialyl-Tn (STn) and the T antigen. They result from a premature stop in protein *O*-glycosylation and are classically termed simple mucin-type *O*-glycans, reflecting their overexpression in cancer-associated mucins [68]. Nevertheless, these alterations can also be significantly observed in other densely *O*-glycosylated proteins of relevant importance in bladder cancer, namely CD44 and different types of integrins [69, 70]. Several reports attribute the expression of simple mucin-type *O*-glycans to a disorganisation of secretory pathway organelles in cancer cells, mutations on *Cosmc*, a gene encoding a molecular chaperone of T-synthase [16, 71], and absence or altered expression and/or activity of glycosyltransferases [72]. In particular, the overexpression of *ST6GalNAc-I* has been found to promote the premature sialylation of the Tn antigen and consequent formation of the STn antigen in bladder cancer [64, 69]. Specifically, the STn antigen is absent in the healthy urothelium, while being present in more than 70% of high-grade NMIBC and MIBC, denoting a cancer specific nature [64]. This post-translational modification of cell surface proteins is mostly expressed in non-proliferative tumour areas, known for their high resistance to cytostatic agents currently used to improve the overall survival of advanced stage bladder cancer patients [64]. Recently, a novel STn-dependent mechanism for chemotherapeutic resistance of gastric cancer cells to cisplatin has been described, in which STn protects cancer cells against chemotherapeutic-induced cell death by decreasing the interaction of cell surface glycan receptors with galectin-3 and increasing its intracellular accumulation [73]. Nevertheless, the relationship between chemoresistance and STn overexpression remains to be fully explored in bladder cancer. Furthermore, STn expression is significantly higher in MIBC when compared to NMIBC, denoting its association with muscle invasion and poor prognosis [20]. Studies *in vitro* have further demonstrated that this antigen plays an important role in bladder cancer cell migration and invasion through mechanisms so far unexplored [64, 69]. Recent glycoproteomics studies of bladder cancer cell models highlighted that STn was mainly present in integrins and cadherins, further reinforcing a possible role for this glycan in adhesion, cell motility and invasion [69]. Also, recent work from our group has demonstrated the presence of STn in lymph node and distant metastasis, strengthening the notion that STn expression may influence cancer cell motility and metastization (unpublished data). Furthermore, STn-expressing bladder cancer cells have shown the ability to induce a tolerogenic microenvironment by impairing dendritic cells maturation, allowing cancer cells to evade innate and adaptive immune system responses [21]. Interestingly, the tolerogenic effect of short-chained *O*-glycans has also been correlated with bladder tumour metastasis through a mechanism in which MUC1 carrying core 2 *O*-glycans functions as a molecular shield against NK cells attack, thereby promoting metastization [74]. In addition, STn expression in bladder cancer tissues has been used in combination with other surrogate markers of tumour aggressiveness envisaging patient stratification regarding disease stage and therapeutic benefit. Specifically, expression of STn and sialyl-6-T (s6T), a sialylated form of T antigen, are independent predictive markers of BCG treatment response and were found useful in the identification of patients who could benefit more from this immunotherapy [75]. Moreover, STn was found to be a marker of poor prognosis in bladder cancer and, in combination with PI3K/Akt/mTOR pathway evaluation, holds potential to improve disease stage stratification [20]. In turn, it was observed that the reduction of Tn antigen expression was associated with higher bladder cancer stage [67].

Several reports associated the presence of T antigens with higher grade, stage and poor prognosis in bladder cancer [66, 76], suggesting that these antigens may be surrogate markers of profound cellular alterations. Also, there is growing evidences linking the overexpression of ST3Gal.I, the enzyme responsible for T antigen sialylation, with higher stage and poor prognosis [65]. Moreover, the expression of T antigen is significantly associated with higher risk for subsequent recurrences with deep muscle invasion and metastatic involvement of regional lymph nodes [67]. In agreement with these observations, we have recently reported that short-chain *O*-glycans are preferentially accumulated in hypoxic tumour areas [69], known to harbor more malignant sub-populations. It has been suggested that HIF-1α directly or indirectly modulates the expression of glycosyltransferases involved in the initial steps of *O*-glycosylation while repressing core elongation, thereby promoting an accumulation of precursor structures [69]. The fact that these simple glycans are absent, significantly under-expressed or restricted to some cell types in healthy tissues, makes them ideal diagnostic and therapeutic targets for bladder cancer therapy [77].

#### Overexpression of cancer-associated membrane glycoproteins

Alteration in *N*- and *O*-glycosylation and other types of protein glycans are often amplified in cancer cells by the overexpression of key cancer-associated glycoproteins. Namely, HER2 (also known as ErbB2 or HER2/neu) is an heavily glycoprotein [78], member of the EGF receptor (EGFR) family, that is overexpressed in several malignancies, including advanced stage bladder cancer [79-81]. Curiously, the incidence of HER2 overexpression in bladder cancer (12.4%) is even higher than that found in breast carcinomas (10.5%), where it is associated with tumour aggressiveness, prognosis and responsiveness to therapy [81]. In fact, HER2 expression is also associated with poor prognosis in bladder cancer [82]. Thus, HER2 could serve as a useful biomarker for clinical prediction and trials of anti-HER2 agents are warranted in patients with advanced bladder cancer. Nevertheless, the glycosylation of HER2 in bladder cancer remains to be addressed, which would be critical for the establishment of a more sensitive and specific biomarker.

EpCAM, also known as CD326, is a glycoprotein predominantly located in intercellular spaces of epithelial, progenitor and normal stem cells [83, 84]. This transmembrane macromolecule regulates both normal and cancer-associated cellular adhesion, proliferation, differentiation, migration and invasion [84, 85]. Its expression is associated with increased tumour stage and grade, as well as with poor prognosis and decreased overall survival in bladder cancer patients [86, 87]. Despite these evidences, the glycosylation pattern of EpCAM in bladder cancer has also not yet been evaluated.

Frequently, cancer cells also overexpress galectins, *N*-acetyllactosamine-binding glycoproteins yielding either one or two carbohydrate-recognition domains. Galectins cross-link glycoproteins depending on their glycan structures and concentrations, forming galectin-glycan molecular lattices [88]. Particularly, the correlation between increased galectin expression and tumour progression is proposed to be linked to their interaction with poly-*N*-acetyllactosamines on matrix proteins such as laminin, aiding cellular invasion [89]. Moreover, these glycoproteins are known to modulate cell growth, differentiation, adhesion, and apoptosis [90-92]. The altered expression of galectins has been implicated in bladder cancer malignancy [93], and both galectin-1, -2, -3, and -8 were suggested as potential disease markers and possible targets for bladder cancer therapy [94]. Specifically, galectin-1 is a possible independent prognostic marker of urothelial carcinoma [95], with its positive immuno-expression being significantly correlated with tumour stage, grade, vascular invasion and nodal status [96]. Moreover, galectin-1 mRNA and protein levels are markedly increased in most high-grade bladder tumours compared with low-grade and normal bladder tissue [97, 98]. Furthermore, this glycoprotein is associated with bladder cancer cell invasion by mediating the activity of MMP9 through the Ras-Rac1-MEKK4-JNK-AP1 signalling pathway [95]. Recently, a photodynamic therapeutic approach targeting galectin-1 in bladder cancer cells and xenografts has inhibited tumour growth and enabled selective cytotoxicity in cancer cells, preventing undesired phototoxicity in the surrounding healthy tissues [99]. This study ultimately suggests that galectin-1 constitutes a valid bladder cancer cell biomarker capable of being used in effective targeted therapies. In turn, galectin-3 mRNA and protein levels were also found increased in bladder tumours when compared with normal urothelium [94, 97, 98, 100]. Moreover, galectin-3 levels are increased in invasive tumours compared with non-muscle invasive lesions [101-103]. Furthermore, its expression patterns are also correlated with tumour stage, grade, proliferation (Ki67), apoptosis (apopdetek and bcl-2), and overall survival in patients with T1G3 tumours [101]. These observations suggest a role for galectin-3 as a biomarker for bladder cancer staging and prognosis. In succession, galectin-7 was pointed as a predictive marker of chemosensitivity to cisplatin in urothelial cancer [104]. Finally, the loss of galectin-8 in bladder tumours increases tumour recurrence, while decreased immunohistochemical staining is associated with higher tumour stage and grade [105]. As such, the loss of galectin-8 might be an early step in the development of malignant lesions of the bladder and is a significant independent predictor of recurrence [105].

Several studies have recently pointed out the unique biological properties of basal-like bladder tumour cell subpopulations in their anchorage-independent growth ability and their association to poorly differentiated bladder cancer [106]. In this context, CD44, a member of the transmembrane glycoprotein family commonly implicated in cell-cell and cell-matrix interactions, cell proliferation, differentiation, migration, angiogenesis, presentation of cytokines, chemokines, and growth factors to the corresponding receptors, docking of proteases at the cell membrane, and cell survival [107-109], has been implicated as a cancer stem cell (CSC) marker in several malignancies [110-114]. Particularly, both CD44 and its splicing variants have been involved in bladder cancer carcinogenesis and progression. CD44+ cells exhibit an enhanced capacity to form xenografts in immunocompromised mice as well as chemoresistance compared to CD44− cells [115, 116]. CD44v6, a CD44 isoform containing the CD44v6 exon, has also been shown increased in bladder CSCs [117, 118]. CD44v6 expression on CSCs is supported by a study that correlates CD44v6 expression on bladder cancer cell lines with stem cell properties [119]. Both expression levels of CD44 and CD44v6 were higher in invasive bladder tumours than in pre-invasive tumours and normal urothelium [120]. Also, CD44 and CD44v6 upregulation is associated with higher tumour grade and stage [120, 121]. However, other studies have demonstrated an inverse association between CD44v6 expression and bladder cancer grade and stage [121, 122]. Moreover, the loss of CD44v6 expression was demonstrated as an independent factor for increased recurrence and shorter overall survival [123]. Also, the loss of CD44 expression was associated with shorter progression-free survival [124]. These discrepancies can be explained by the lack of standard immunohistochemical assays, the use of antibodies with different specificities, and differences in the clinicopathological status of bladder tumours used in the different studies. Therefore, integrative and standardized studies are necessary to elucidate the role of CD44 and CD44v6 in bladder cancer, as they hold an important biological and clinical value and may serve as therapeutic targets. In turn, CD44 variant 9 (CD44v9) overexpression has been associated with shorter progression-free and cancer-specific survival in bladder cancer [125], likely impacting invasion and migration via the epithelial-mesenchymal transition (EMT). Therefore, its expression might be a useful predictive biomarker in basal-type muscle invasive and high-risk NMIBC [125]. Nevertheless, the specific glycosylation patterns of CD44 in the context of bladder cancer also remains an open research topic.

Mucins are large membrane-bound glycophosphoproteins, commonly overexpressed in several malignancies [126], including bladder cancer [127-129]. Mucin 1 (MUC1) is restricted to the apical membranes of umbrella cells in normal urothelium, while there is an aberrant MUC1 expression in basal and intermediate layers of neoplastic epithelium [128, 130]. Additionally, the pattern, intensity and depth of MUC1 immunostaining are correlated with bladder cancer grade [129]. Notwithstanding, other study reported no correlation of MUC1 expression with survival, tumour stage or grade [131]. Yet, patients overexpressing MUC1 only had a favourable survival when HER3 was also overexpressed [131]. This may be at least partially explained by the existence of several MUC1 glycoforms, including underglycosylated, sialylated, and fully glycosylated forms. As previously mentioned, several studies have been focusing on the identification of extracellular cell surface markers for urothelial CSCs, envisaging diagnosis and drug targeting. Of note, it has been shown that urothelial CSCs are enriched in an MUC1−CD44v6+ subpopulation of cells. This conclusion was based on the observation that MUC1− and CD44v6+ cells were only present in the basal layer of normal urothelium, which is thought to comprise urothelial stem cells. Subsequently, MUC1− and CD44v6+ cells were isolated, and a slightly increased clonogenicity was observed for these cells compared with unsorted bladder tumour cells [117]. Expression of other mucins such MUC2 and MUC6 were associated with a less aggressive behavior of bladder tumours and demonstrated to be useful predictors of better bladder cancer survival while MUC4 demonstrated an opposite role [129]. In addition, MUC16 STn+ glycoforms, characteristic of ovarian cancers, were recently described for the first time in bladder cancer and demonstrated to be expressed in a subset of advanced-stage bladder tumours facing worst prognosis [132]. Nevertheless, with the exception of MUC16, the specific glycosylation patterns of this class of glycoproteins also remains unknown in bladder cancer.

Integrins are a family of transmembrane adhesion receptors for extracellular matrix components participating in the metastatic cascade. Particularly, normal urothelium presents a polarized expression of alpha6beta4 integrin (ITGA6) on basal cells, while neoplastic urothelium frequently overexpresses this receptor [133]. Moreover, the evaluation of alpha6beta4 integrin tumour expression may provide valuable prognostic information on bladder cancer patients clinical outcome, since patients with alpha6beta4 integrin overexpression hold a significantly worst survival [133]. Throughout EMT-driven carcinogenesis, disseminated cancer cells often acquire a stem cell-like self-renewal capability [134, 135]. Moreover, during EMT, epithelial markers such as ITGAV (αv integrin receptors) are upregulated in several solid tumours [136-138], including bladder cancer with a trend increase in ITGAV expression with disease stage and grade [139]. Furthermore, the functional inactivation of ITGAV (targeting with the integrin receptor antagonist GLPG0187 or knockdown of ITGAV) leads to a less malignant bladder cancer phenotype with significantly impaired migration, EMT response, clonogenicity and a reduction in the size of the stem/progenitor pool. In line with these *in vitro* observations, knockdown of ITGAV or treatment with GLPG0187 significantly inhibited metastasis and secondary tumour growth [140]. In turn, a central role was also suggested for the beta1-integrin subunit in forming the cell-cell and cell-matrix bonds necessary for adhesion, extravasation and migration of bladder cancer cells [141] through enhanced transmission and generation of contractile forces [142] and possible microenvironmental involvement [69]. Despite its role in bladder carcinogenesis there are also no reports about the specific glycosylation of this class of glycoproteins.

In summary, increased levels of several glycoproteins have been associated with the severity of disease and as part of the molecular signature of more malignant bladder cancer sub-populations. These events not only amplify structural alterations that stem from deregulations in glycosylation pathways but also synergically contribute together with altered glycosylation, to a net effect favouring disease progression. Nevertheless, a comprehensive and context-oriented glycomapping of relevant glycoproteins has not been provided yet, which would be crucial for achieving highly specific cancer biomarkers holding true therapeutic potential. Moreover, the glycomic mapping of relevant glycoproteins may provide highly cancer-specific epitopes in comparison to glycans or glycoproteins alone. This would pave the way for designing more effective targeted therapeutics for more malignant bladder cancer cells.

### Proteoglycan glycosylation

Proteoglycans are structurally and functionally complex glycoconjugates, exhibiting one or more high molecular weight glycosaminoglycan (GAG) chains covalently attached to a protein core [143]. These structures can be found as: i) transmembrane syndecans or glypicans, at the cell surface; ii) hyalectans (aggrecan, versican, brevican and neurocan) or small leucine-rich proteoglycans (decorin, biglycan and lumican) at the extracellular matrix (ECM); iii) basement membrane proteoglycans (perlecan, agrin and collagen XVIII) [144]. Serglycin is the only characterized proteoglycan found at intracellular level, normally in secretory compartments [145].

The biosynthesis and modification of proteoglycans occurs in the Golgi apparatus (GA) through the action of glycosyltransferases, sulfotransferases, epimerases, sulfatases, glycosidases, and heparanases, revealing multiple layers of regulation of these macromolecules [143]. The length and structure of each GAG chain may differ greatly within a certain proteoglycan molecule, while the number of chains linked to the protein core is determined by the number of sugar attachment sites, marked by Ser-Gly dipeptide motifs [143, 146]. The biosynthesis of GAGs, such as chondroitin sulfate, heparan sulfate, dermatan sulfate, hyaluronic acid, and heparin is initiated by the sequential addition of four monosaccharides (Xyl, Gal and GlcA) to a Ser-Gly motif on the core protein. Then, the sugar chains are extended by the addition of two alternating monosaccharides containing an acetylated or sulfated hexosamine (GalNAc, GlcNAc) and uronic acid (GlcA acid or idoA) [143]. In the case of keratan sulfate, the GAG is initiated as *N*-linked or *O*-linked repeating disaccharides, and extended by the addition of *N*-acetyl-glucosamine and galactose residues [143]. Once synthesized, the GAGs are linked to a core protein and proteoglycans are transported from the GA to the cell surface or ECM [144, 147]. Notably, unlike all other GAGs, hyaluronic acid is primarily found as a free sugar chain at the ECM, and its synthesis is epigenetically regulated [148]. Interestingly, hyalectans have the ability to bind hyaluronic acid through their N-terminal globular domain (G1), therefore increasing ECM complexity [149]. Of note, proteins such as MHC class II invariant chain, transferrin receptor, thrombomodulin and CD44 can be considered proteoglycans, since some of their alternative splicing variants present GAG-initiation sites [150]. Other proteoglycans like endocan and versican also present alternatively spliced forms with variable sugar modifications [150]. In particular, a versican variant without chondroitin sulphate attachment sites has been described, [149].

**Figure 4:**
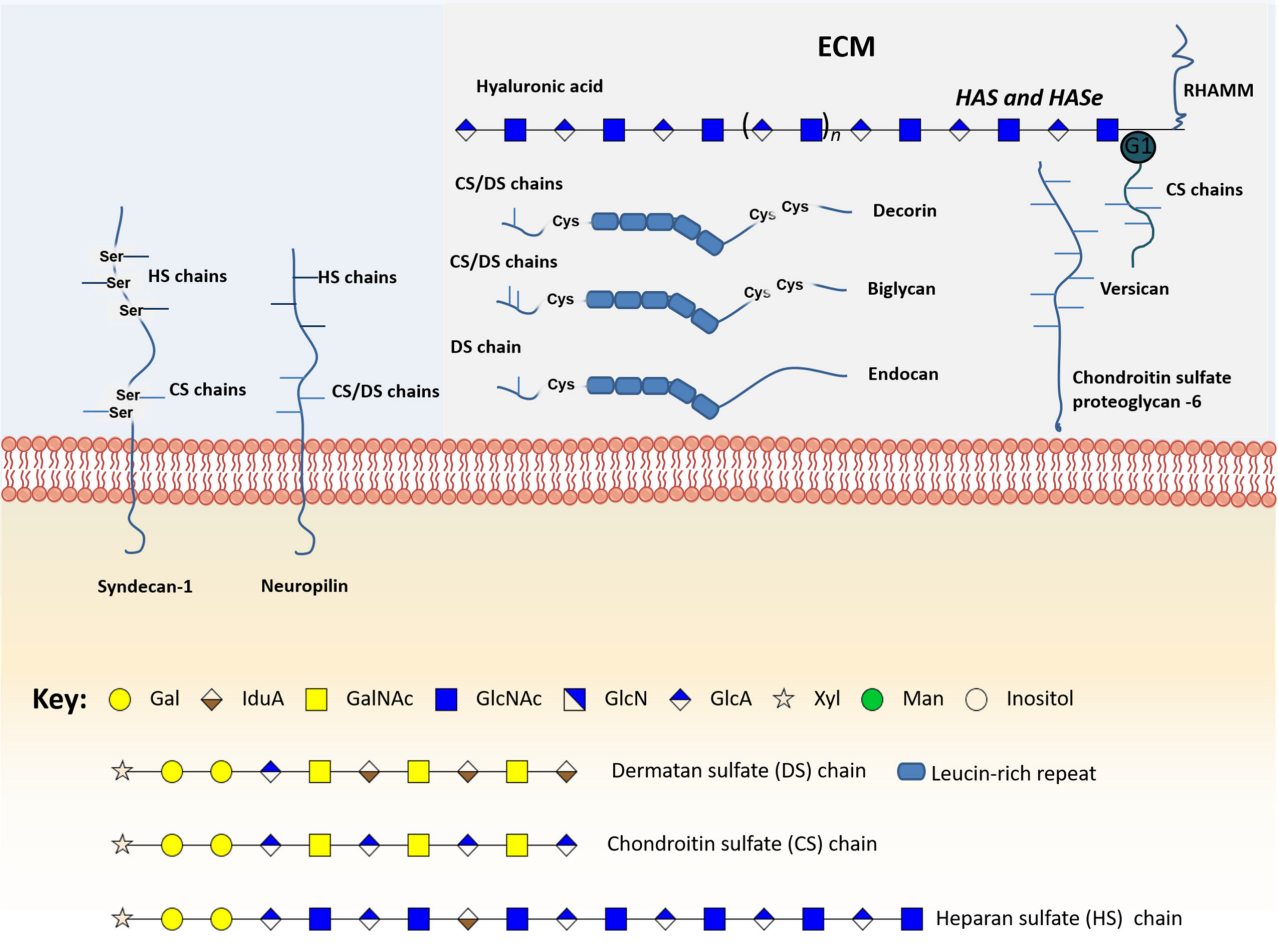
Schematic representation of the main glycomolecules with biological relevance in bladder cancer The figure represents specific proteoglycans that have one or more glycosaminoglycan (GAG) chains, consisting of linear co-polymers of acidic disaccharide repeating units such as chondroitin sulfate (DS), heparan sulfate (HS) and dermatan sulfate (DS). These glycomolecules can be found attached to the outer leaflet of the plasma membrane (Syndecan-1) or in the extracellular matrix (Versican, Chondroitin sulfate proteoglycan-6, decorin and biglycan). Particularly, some of these structures can bind to each other through their N-terminal globular domain (G1), therefore increasing extracellular matrix (ECM) complexity. Of note, hyaluronic acid is the only GAG primarily found as a free sugar chain in the extracellular matrix. Hyaluronic acid synthases (HAS) and Hyaluronidases (HAse) constantly degrade and remodel hyaluronic acid molecules largely affecting ECM dynamics. Some glycoproteins can also be found linked to the cell membrane through a glycosylphosphatidylinositol (GPI) anchor, an example is glypican-3.

Proteoglycans present high affinities for various ECM constituents and cell adhesion molecules, playing a crucial role in intercellular interactions [144]. These glycoconjugates can also bind growth factors, cytokines and chemokines, allowing them to escape proteolysis. Some can also act as co-receptors for growth factors and tyrosine kinase receptors, changing the duration of their signaling reactions or lowering their activation thresholds [143, 144]. Therefore, the altered expression of proteoglycans, including syndecan-1, neuropilins, versican, chondroitin sulfate proteoglycan 6, decorin, biglycan, endocan, hyaluronic acid and its metabolic enzymes, has been linked to several cancers and, specifically, with bladder cancer carcinogenesis, metastasis and prognosis (Figure 4, Supplementary Table 1).

#### Cancer-associated transmembrane proteoglycans

Syndecans are a family of heparin sulfate proteoglycans, commonly presenting three to five heparin sulfate chains, and are known to modulate cellular adhesion, migration, proliferation, differentiation, and growth factor signaling [151]. These macromolecules are commonly found at bladder cancer cell surfaces, along with other transmembrane proteoglycans and glypicans [152, 153]. Syndecans can also be found in their soluble form, due to a post-translational modification causing the release of their ectodomains through juxtamembrane region proteolysis [154]. Particularly, syndecan-1 (CD138), frequently expressed in epithelial cells and some leukocytes [155], was found to be increased in bladder cancer patients serum and stroma, especially in muscle-invasive cases [156-158]. Serum overexpression of syndecan-1 was associated with lymph node metastasis, while stromal overexpression was related with poorer overall survival [158]. The loss of transmembrane syndecan-1 expression in tumour cells was related to higher tumour stage and grade [159, 160], as well as reduced recurrence-free survival in bladder cancer [160, 161]. Still, high-grade superficial, and deep invasive bladder carcinomas were also characterized by elevated expression of syndecan-1, while low-grade and non-invasive phenotypes do not [161]. Cytoplasmic overexpression of syndecan-1 in cancer cells, often accentuated close to the nucleus, was demonstrated in Ta tumours compared to normal urothelium, suggesting a failure in intracellular trafficking caused by the loss of functional syndecan-1 [162]. This directly affects carcinogenesis through the loss of cellular adhesion properties, thereby promoting more invasive phenotypes [160]. Also, syndecan-1 altered expression can affect tumour cells via junB-FLIP long signals, involving apoptosis resistance and increased proliferation [161]. Simultaneous loss of syndecan-1 expression in tumour cells and its overexpression in high-stage and high-grade bladder cancer patients serum suggest the importance of syndecan-1 in tumour progression; therefore, this molecule could be a new therapeutic target in human urinary bladder cancer [158].

Neuropilins are co-receptors of two structurally and functionally unrelated ligands classes, the class 3 semaphorins and selected VEGF family members [163]. Neuropilin-1 has multiple heparan and/or chondroitin/dermatan sulfate GAG chains [163]. Recent reports demonstrate neuropilin-1 expression on non-endothelial cells in bladder urothelium [164], as well as its overexpression in high grade/stage bladder tumours [165]. Moreover, neuropilin-1 upregulation was associated with shorter overall survival in bladder cancer patients [165]. In addition, neuropilin-2 is expressed in neural and endothelial cells and, upon ligand stimulation, induces neural development and the growth of newly formed blood and lymphatic vessels [163]. Overexpression of neuropilin-2 demonstrated to have prognostic value in bladder cancer, as it was associated with shorter overall and cancer-specific survival and earlier cancer-specific death after transurethral resection and radiochemotherapy [166]. Additionally, the co-expression of neuropilin-2 and the family member VEGF-C is also a prognostic marker for overall survival of bladder cancer patients [166]. Therefore, syndecan-1 and neuropilins may play an important role in the progression of bladder cancer and their altered expression may serve as a biomarker for prognosis.

#### Cancer-associated extracellular matrix proteoglycans

Versican, also known as chondroitin sulfate proteoglycan 2, a central component of cancer-related inflammation, is highly expressed in metastatic bladder carcinomas and its overexpression is correlated with poor survival [167]. In tumour cell lines, versican overexpression was associated with increased cell migration and tumour stage [168]. A correlation between versican overexpression, RhoGTP dissociation inhibitor 2 (RhoGDI2) underexpression, metastasis and poor clinical outcome was also demonstrated [167, 169]. Particularly, RhoGDI2 underexpression and versican overexpression are associated with metastasis through the involvement of macrophages and the CCL2/CCR2 signaling axis [167, 169]. In fact, RhoGDI2 is a regulator of several Rho GTPases that play important roles in cell cycle progression, neovascularization, invasiveness, and metastasis [170]. Therefore, targeting this mechanism may provide novel therapeutic strategies for delaying the appearance of clinical metastasis [170].

The role of decorin, a key component of the tumour stroma, in cancer progression and its therapeutic potential has been the focus of several studies. Increased secretion of decorin in the MB49/MB49-I murine bladder cancer model and in muscle-invasive tumours was associated with the promotion of angiogenesis and tumour cell invasiveness [171]. Nevertheless, other studies demonstrate a possible tumour suppressor role for decorin, where bladder tumour tissues are entirely devoid of decorin expression while non-malignant stromal areas express this proteoglycan [172, 173]. A mechanism through which decorin exerts its tumour suppressor role has been proposed, where decorin may act as a natural antagonist of the oncogene insulin-like growth factor receptor I (IGF-IR) [173, 174]. Therefore, in bladder tumours, the loss of decorin expression eliminates IGF-IR activity and signaling repression, promoting cellular motility, invasion, and cancer progression [173, 174].

Biglycan is a small leucine-rich proteoglycan with immune and growth factor activity modulating properties, as well as matrix assembly involvement [175]. This proteoglycan has been demonstrated to be overexpressed on invasive bladder cancer tissue [172, 176]. Interestingly, while biglycan overexpression is associated with higher tumour stages and muscle invasiveness, it’s up-regulation was related with tumour cell proliferation inhibition and increased patients’ 10-year survival [176].

Endocan, also known as endothelial cell-specific molecule 1, is a secreted proteoglycan that has a single dermatan sulfate side chain attached to serine 137 and demonstrated to be highly elevated on tumour vessels from invasive bladder cancer tissues [177]. Moreover, its expression correlated with stage, and invasiveness as well as predicted a shorter recurrence-free survival time in non-invasive bladder cancers [177]. Therefore, endocan expression impacts the prognosis of bladder cancer patients and, as described ahead, also is a possible diagnosis marker.

Hyaluronic acid (HA), an unsulfated anionic linear GAG, its implicated in cell adhesion, migration and angiogenesis [178]. Particularly, hyaluronidases (HAse) are enzymes that hydrolyze HA molecules into small angiogenic fragments, participating in the degradation of tumour surrounding ECM, and enabling cancer cells invasion and dissemination [178]. As such, HA, HAses (e.g. HYAL1), hyaluronic acid synthases (HAS) 1, 2 and 3, as well as hyaluronic acid receptors (e.g. CD44 and receptor for hyaluronan-mediated motility, RHAMM) have been suggested as possible diagnosis and prognosis biomarkers. Also, both HAS, HYAL1, CD44 and RHAMM were found to be overexpressed in bladder cancer tissues [179, 180]. In addition, HYAL1 expression was also correlated with disease-specific mortality and recurrence [179, 181]. Finally, elevated expression of RHAMM was found in invasive bladder tumours and associated with poor prognosis, due to increased tumour cell proliferation and shorter overall and disease-specific survival [180].

Alterations in cancer-associated proteoglycans were demonstrated as associated with bladder cancer progression and may present prognosis value, yet more studies are necessary in order to confirm these associations and transpose these markers to clinical practice.

### Lipid glycosylation

Glycolipids are a major class of glycoconjugates that include glycosphingolipids (GSLs) and GPI anchors.

#### Alterations in sphingolipids’ glycosylation

Glycosphingolipids (GSLs) are neutral or anionic molecules composed by a hydrophilic glycan covalently β-linked via glucose (glucosylceramide) or galactose (galactosylceramide) to the terminal hydroxyl group of a hydrophobic ceramide backbone [182, 183]. Specifically, GSL biosynthesis is initiated in the ER with the condensation of sphingosine and acyl-CoA by a group of six ceramide synthases, giving rise to a long-chain amino alcohol base (sphingosine) in amide linkage to a fatty acid, namely a ceramide lipid [183, 184]. Ceramide can then be galactosylated by galactosylceramide synthase, to produce galactosylceramide, which in turn can be transported to the GA where it is sialylated to produce GM4 ganglioside, or sulfated to produce sulfogalactolipids [185-187]. Also, ceramide is frequently glucosylated in the GA by glucosylceramide synthase to form glucosylceramide, the core structure of 90% of GSLs [185-187]. Subsequently, the C-4 hydroxyl of glucosylceramide can be galactosylated by β4-galactosyltransferases V and VI, forming lactosylceramide [188]. Once produced, lactosylceramide will serve as the metabolic precursor of more than 300 structurally different classes of complex GSLs through the action of specific glycosyltransferases and sulfotransferases, depending on nucleotide sugar donors availability [189]. Particularly, lactosylceramide is a template for: 1) GA2, through β,4-N-acetylgalactosylaminyltransferase (B4GALNT1) activity; 2) GM3 ganglioside, through α-2,3-sialyltransferase (ST3GAL5Gb3); 3) Gb3, by α-1-4-galactosyltransferase (A4GALT) activity; and, 4) Lc3, by the β-1,3-N-acetylglucosaminyltransferase (B3GNT5) [189]. GSLs glycan chains can be further extended and terminated with structural moieties similar to those found in glycoproteins, namely and Lewis blood type antigens. After synthesis, these structures leave the GA and are redirected to the plasma membrane [190], constituting approximately 5% of all membrane lipids

**Figure 5:**
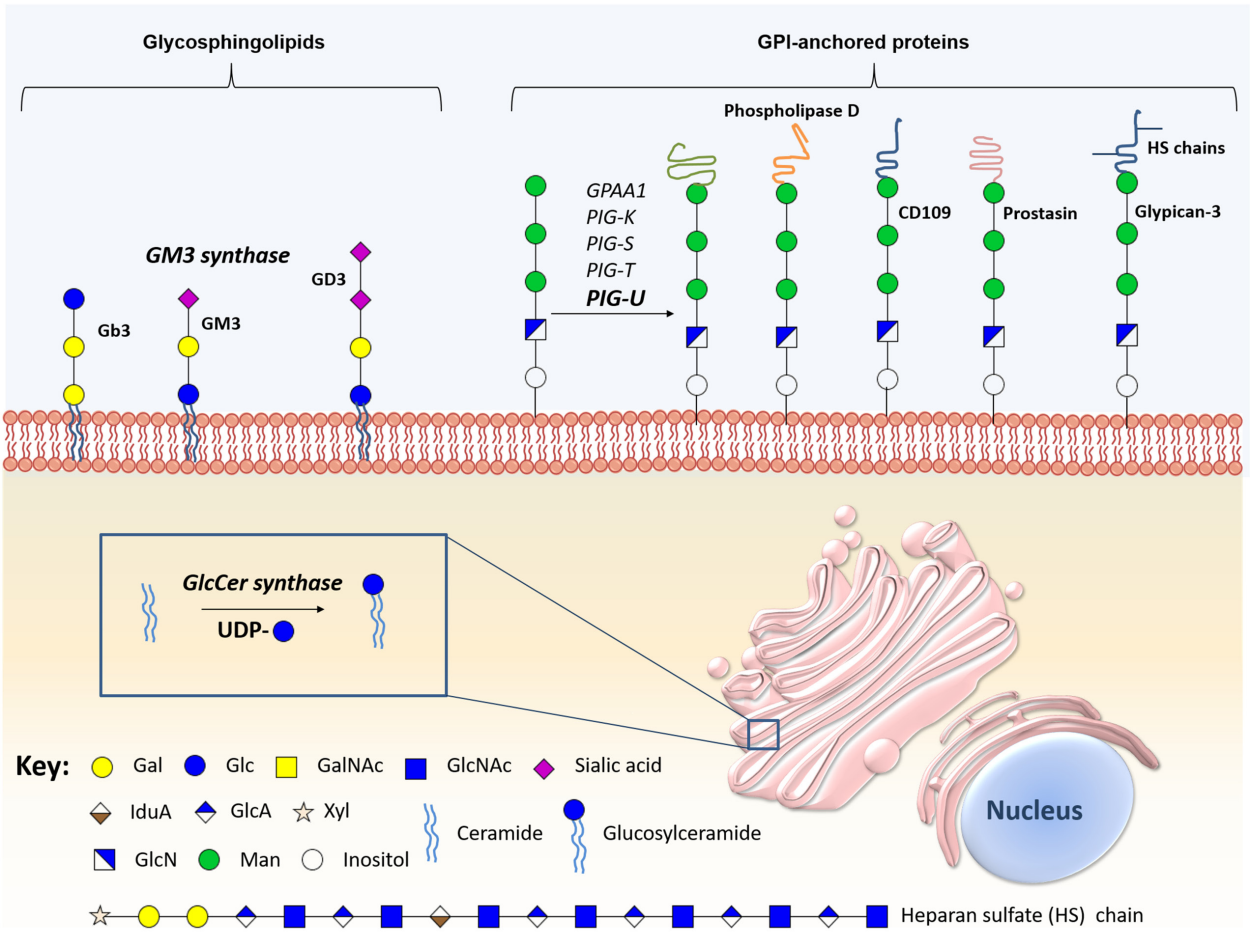
Schematic representation of the main biologically relevant glycosphingolipids and glycosylphosphatidylinositol-anchored proteins in bladder cancer The figure represents certain glycosphingolipids, especially the sialic acid-containing glycomolecules, glycosylphosphatidylinositol-anchored proteins, and the enzymes implicated in the synthesis and hydrolysis of these conjugates have been implicate in bladder cancer malignancy.

GSLs are also implicated in key cellular functions, such as cell adhesion, proliferation, differentiation, apoptosis, motility and immune recognition [191-193]. Particularly, ceramide is implicated in apoptosis and regulates several cell cycle and senescence pathways [194]. Consequently, GSLs have received considerable attention as promising biomarkers for disease progression, as well as pharmacological targets for bladder cancer therapy. Pioneering studies demonstrated that glucosylceramide and glucosylceramide synthase are overexpressed in several multidrug resistant cancer cell lines, being related with drug resistance [195-197]. Particularly, in bladder cancer, glucosylceramide synthase overexpression was demonstrated to be associated with higher histologic grade [198]. In accordance, the overexpression of this enzyme is an indicator of poor prognosis, showing associations with lymph node metastasis, reduction in the 5-year overall and disease-free survival [198]. The glycosphingolipid composition of human bladder cancer tissue has been assessed, showing large amounts of ganglioside GM3 in superficial bladder tumours, but not in invasive tumours [199]. This overexpression can be caused by the simultaneous overexpression of GM3 synthase and downregulation of both Gb3 and GD3 synthases [199]. Moreover, high levels of GM3 are associated with reduced invasive potential [199], proliferation, motility, tumour growth and increased apoptosis [200]. Since exposure to exogenous GM3 have been proved to inhibit tumour cell lines proliferation and adhesion, this approach was proposed as bladder cancer therapy. Also, the direct instillation of GM3 in orthotopic models inhibited tumour growth [201]. It has also been reported that the expression of GM2, GM3, or GM2/GM3 complexes inhibited bladder cancer cell motility and growth [202] (Figure 5, Supplementary Table 1).

### Alterations in glycosylphosphatidylinositol-anchored molecules

The anchoring of proteins and proteoglycans to cell membranes through the lipid portion of a GPI anchor is a conserved post-translational modification [203]. These anchors present conserved core structures consisting of ethanolamine phosphate, three mannose monomers, and a non-*N*-acetylated glucosamine attached to a inositol phospholipid (EtNP-6Manα-2Manα-6Manα-4GlNα-6myoInositol-P-lipid) [204]. This backbone can be modified with phosphoethanolamine and/or various glycan side-branches. More detailed information about the biosynthesis of this class of glycans may be found in [204-206] and has been summarized in Figure 5.

Alterations in several enzymes involved in glycosylation of GPI-anchored molecules such the multi-protein transaminase complex were first mentioned as relevant in cancer after the discovery of the oncogenic activity of PIG-U in bladder cancer [207, 208]. This was demonstrated through the induction of tumourigenesis mediated by PIG-U overexpression in mice [208]. In addition, PIG-U overexpression *in vitro* was correlated with increased cell proliferation and upregulation of GPI-anchored proteins, such as urokinase receptor, increasing STAT-3 phosphorylation and subsequent cellular migration, and apoptosis [208]. PIG-U overexpression is also associated with higher tumour grade and muscle invasion, suggesting its role in tumour development and progression [209]. Moreover, overexpression of PIG-U is an independent predictor of recurrence for superficial bladder cancer [209]. Consequently, the expression of PIG-U and other multi-protein transaminase complex subunits was explored and confirmed in different cancer types using microarrays [210].

GPI-anchored proteins are almost exclusively located on cell surfaces, and are functionally diverse, presenting key roles in cell-cell interaction, adhesion, host defense, and signaling transduction [204, 211]. Thus, these proteins have been explored regarding their potential as biomarkers for carcinogenesis and metastatic potential in bladder cancer (Figure 5, Supplementary Table 1). The expression of GPI-specific phospholipase D, a highly specific GPI-anchored enzyme, is significantly increased in highly malignant murine bladder carcinoma cells when compared to less malignant controls [212]. In addition, CD109, a GPI-anchored glycoprotein that negatively regulates the transforming growth factor (TGF)-β/Smad signaling *in vitro*, is overexpressed in the basal layer of NMIBC and low-grade tumours. Interestingly, CD109 shows a similar expression pattern to cancer stem cell marker CD44, and its overexpression was associated with better cancer-specific survival [213]. Prostasin, a GPI-anchored serine protease crucial for epithelial differentiation [214] and epidermal growth factor receptor (EGFR) proteolysis [215], was shown to be downregulated in high-grade urothelial bladder cancer cell lines [216]. This loss of expression was associated with EMT, marked by a reduced E-cadherin expression and loss of epithelial morphology, which may have implications in the invasive potential and resistance to anti-EGFR therapy [216]. However, the clinical relevance of prostasin was not yet evaluated in bladder cancer. Notwithstanding, multi-protein transaminase complex subunits and GPI-anchored proteins have potential to serve as markers of tumourigenesis and metastatic capability, being relevant targets for bladder cancer therapy.

Beyond GPI-anchored proteins, alterations in glypicans, a class of GPI-anchored proteoglycans, also have been studied in cancer. Glypicans are a family of GPI-anchored heparan sulfate proteoglycans known to interact with growth factors through heparan sulfate chains [217]. This class of proteoglycans is predominantly expressed during fetal development, being critical to organogenesis [218]. Moreover, glypicans were described in several cancers [219, 220], including bladder cancer. Particularly, glypican-3 is expressed in squamous cell and invasive urothelial carcinomas; however, it is not a good biomarker for diagnosis using tumour tissues [217]. Furthermore, glypican-3 expression was not associated with tumour stage, grade, lymph node metastasis, concomitant CIS, soft tissue surgical margins, disease recurrence or cancer specific mortality after radical cystectomy [221].

In conclusion, alterations in PIG-U and GPI-anchored proteins seem to be promising regarding their prognosis value while GPI-anchored proteoglycans appear to be bad prognosis biomarkers. Notwithstanding, further studies are necessary to evaluate these GPI-anchored molecules and enzymes from multi-protein transaminase complex in bladder cancer biological and clinical context.

## PROGNOSIS GLYCOMARKERS FOR BLADDER CANCER

**Figure 6:**
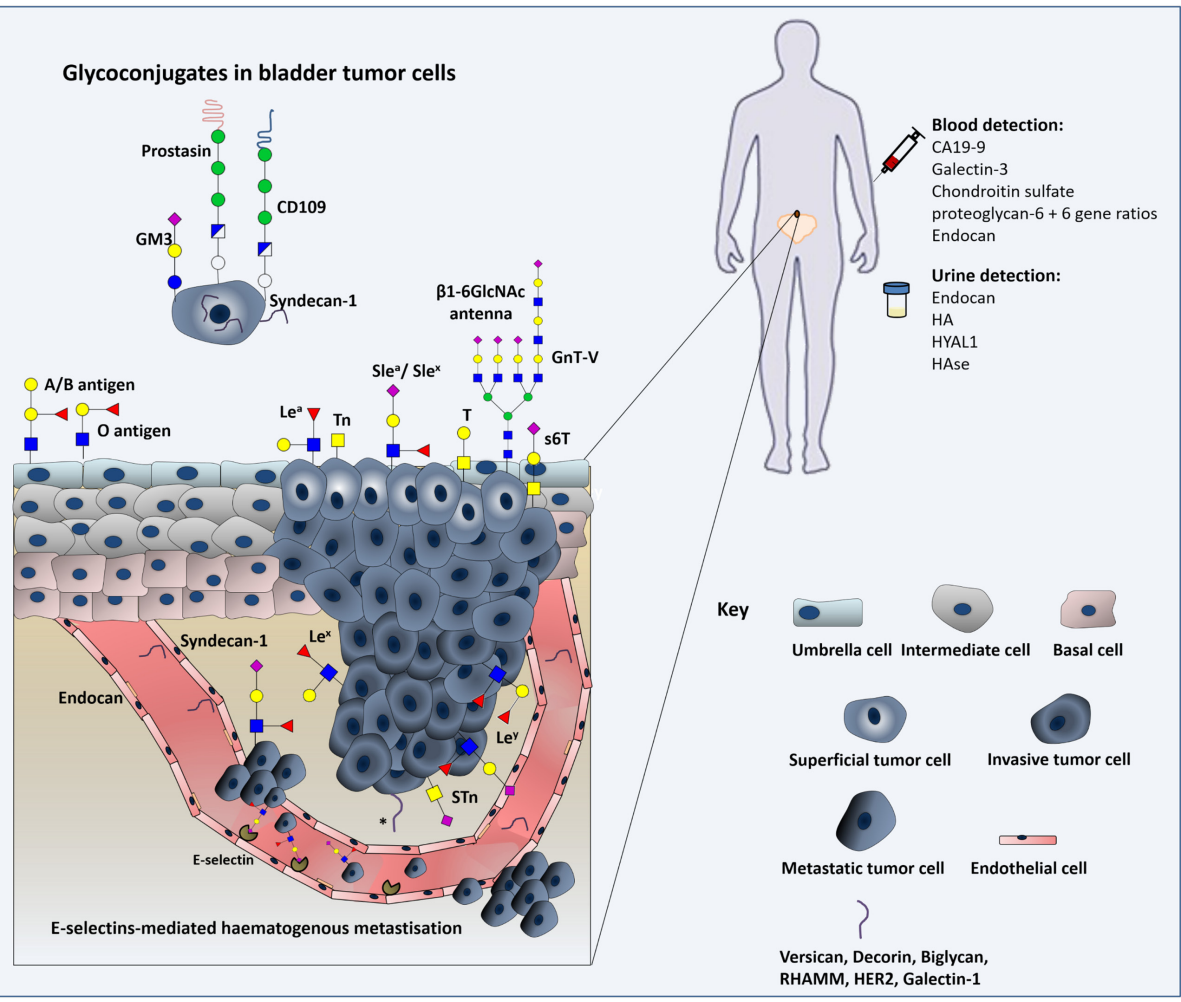
Schematic representation of the glycomolecule-mediated metastization model and diagnostic value of glycans Herein we represent the process of tumour cell invasion, dissociation and metastization in which glycans interfere with cell-cell adhesion and haematogenous tumour cell spread. We emphasize the modification of epithelial cadherin with β1,6-*N*-acetylglucosamine (β1,6GlcNAc)-branched *N*-glycan structures, the loss of ABO(H) blood group determinants, changes in Lewis antigens patterns, and the oversialylation of glycans resulting in the over-expression of simple mucin type O-GalNAc glycans. Furthermore, expression of glycolipids, proteoglycans and gangliosides in cancer cell membranes can modulate signal transduction, activating various cellular pathways that induce tumour growth and progression. As such, some of these relevant glycomolecules are represented as well. The diagnostic value of some of these macromolecules is also highlighted.

**Figure 7:**
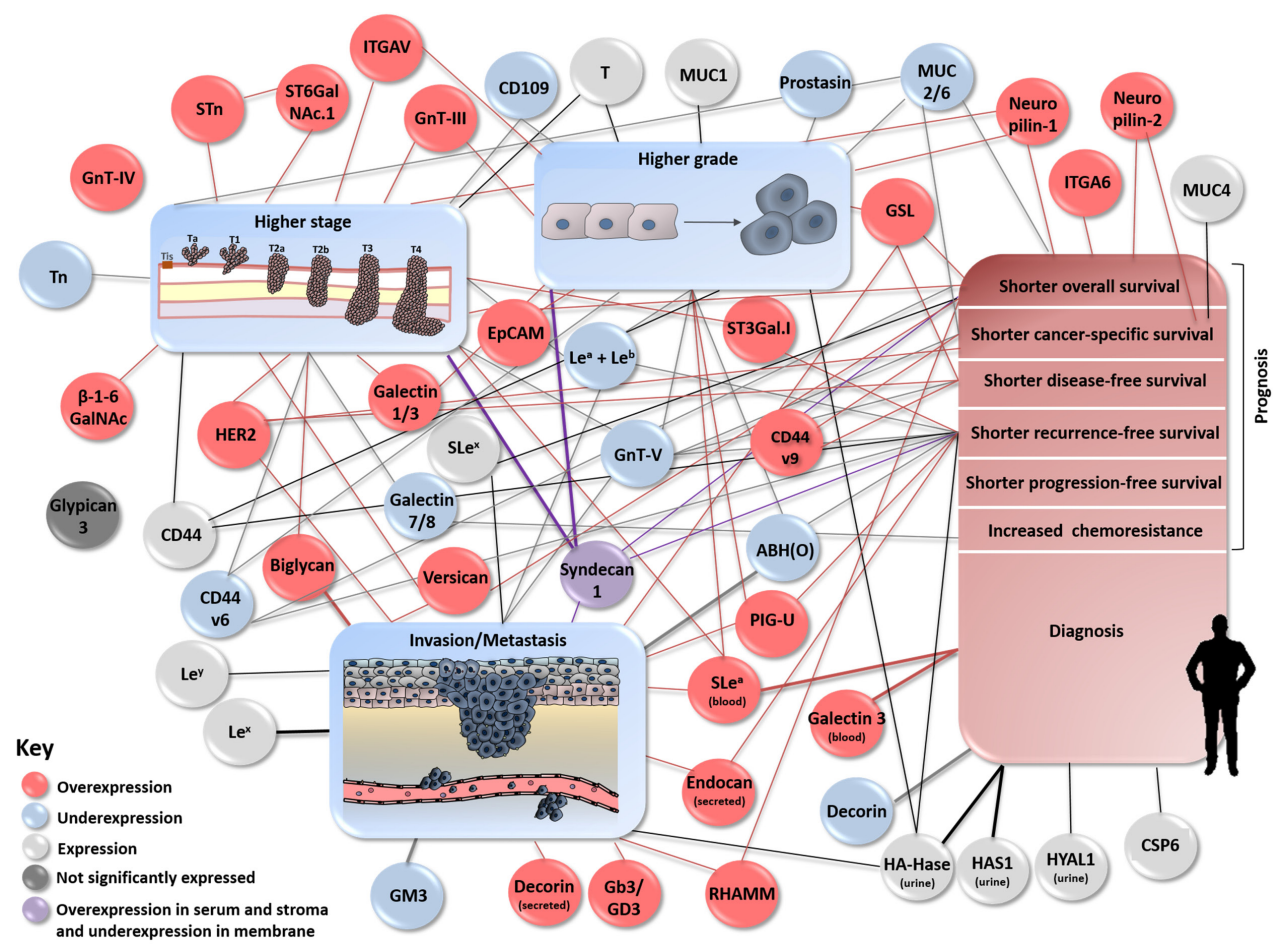
Schematic representation describing associations between (altered) expression of glycans and glycoconjugates and bladder tumour stage, grade, invasion/metastasis, patients’ diagnosis and prognosis. The figure clusters using a gradient of colored circles and lines the biological and clinical role of the altered expression of glycans and glycoconjugates in bladder cancer.

The most challenging aspect in bladder cancer management stems from its highly heterogeneous nature, irrespectively of disease stage. This constitutes a true obstacle for individualized therapeutic decision, disease monitoring and prognosis with tremendously negative impact on patient care and life expectancy. In this context, several clinical studies have unveiled glycan-associated signatures (glycans, glycan binding proteins, glycosyltranserases, heavily glycosylated proteins/lipids/GPI-anchored molecules) associated with more aggressive cancer cell phenotypes, tumour recurrence, progression, metastization, or decreased overall survival. Particularly, in bladder cancer, the presence/overexpression of GnT-III/IV, Le^x^, Le^y^, SLe^x^, STn, ST6GalNAc.I, T-antigen, ST3Gal.I, HER2, EpCAM, galectin-1, galectin-3, CD44, CD44v9, MUC1, MUC4, ITGA6, ITGAV, neuropilin-1, neuropilin-2, versican, decorin, biglycan, endocan, HYAL1, hyaluronic acid synthase 1, RHAAM, glucosylceramide synthase, and PIG-U was associated with more aggressive phenotypes and/or poor prognosis [20, 30, 46, 50, 54, 64-67, 76, 82, 86, 87, 96, 97, 101-103, 120, 121, 124, 125, 129, 133, 139, 165-168, 171, 172, 176, 177, 179-181, 198, 208, 209]. By another hand, (over)expression of β-1-6GalNAc antennae, galectin-7, CD44v6, MUC2, MUC6, glycolipid enzyme GM3, and CD109 were linked to a less aggressive phenotype and/or better prognosis [32, 104, 121, 122, 129, 199, 213]. In turn, the loss of GnT-V, ABO(H) terminal structures, sLe^a^, Tn, galectin-8 and prostasin is associated with more aggressive cancer phenotypes, making them potential markers of poor prognosis [31, 32, 44-47, 50, 67, 105, 158-160, 200, 216]. Yet, glypican-3 tissue expression was not associated with aggressive phenotype nor prognosis [217, 221] and studies for syndecan-1 demonstrated contradictory results [158-162, 222], warranting elucidation of its prognostic and diagnostic role. For other molecules, the co-expression with another allowed to have or enhanced a significant prognostic and therapeutic outcome impact, such as Le^a^/Le^b^ antigens [44, 49], s6T/STn [75] neuropilin-2/VEGF-C [166], Gb3/GD3 synthases [199] and GPI-specific phospholipase D/H-ras oncogene [212] (Figure 6 and 7, Supplementary Table 1).

While these studies support the potential of glycans for patient stratification significant bias hampers conclusive remarks to support introduction in clinical practice. Namely, most studies differ in cohort size and distribution of the cohort by bladder cancer type, stage and grade, which often translate into conflicting results. Moreover, most studies disregard patient ethnicity, lack endpoints standardization and rely on different biological samples, as well as in different sample processing and analysis techniques. Nevertheless, several molecular markers hold promise for becoming widely available and cost-effective tools for a more reliable risk assessment. Thus, efforts need to be conducted to validate glycobiomarkers in larger series for prospective use, ideally in the context of prospective clinical multicenter randomized trials, using current clinicopathological parameters for risk assessment. The inclusion of relevant glycobiomakers in current patient stratification parameters may help accurately assess patient’s prognosis and response to different treatment options. Therefore, future studies should also evaluate the impact of glycomarkers in the therapeutic outcome and as novel targets for bladder cancer therapy. Finally, recent evidences support the need to integrate this knowledge in multiplex risk assessment tool combining standard clinicopathological factors with molecular markers [223] envisaging individualization of responses. Although much effort has been spent on glycobiomarkers research, clinicians and medicinal chemists rarely consider glycans as biological targets or drugs [224], hindering advances in bladder cancer management and in the development of targeted therapeutics. Notwithstanding, this unfamiliarity is beginning to change as improved methods for carbohydrate synthesis [225-227], sequencing [228, 229], and biological analysis [230-232] become more sensitive and widely available.

## EXPLORING GLYCANS FOR NON-INVASIVE BLADDER CANCER DETECTION

Non-invasive disease detection remains a major challenge despite the pressing need for tools capable of reducing the burden of disease-follow up, treatment monitoring and early detection [233]. The active secretion of cancer-associated glycoconjugates into bodily fluids, such as urine and blood, or shed from apoptotic and necrotic cancer cells holds tremendous potential to address this challenge [7, 51]. Accordingly, several FDA-approved cancer biomarkers for non-invasive cancer detection, follow-up and prognosis are either glycans, such as CA19-9 (SLe^a^), or heavily glycosylated glycoproteins, such as CA125 (MUC16), CA15-3 (MUC1), CA-72-4 (tumour-associated glycoprotein 72, TAG-72), PSA (prostate-specific antigen), and CEA (carcinoembryonic antigen) [233]. In bladder cancer, serum CA19-9 is a marker of aggressiveness and advanced stage disease, being almost invariably raised in patients with metastatic cancer. As such, it constitutes a valuable marker of poor prognosis [234]. Notwithstanding, its value as a screening tool has been opposed by its low sensitivity (29%) [234]. On the other hand, urinary CA19-9 is a better screening parameter, with optimum sensitivity and specificity, than its serum counterpart for diagnosis of low grade and early stage bladder cancer. Furthermore, it can be suggested that urinary CA19-9 can be used as better prognostic marker for low grade bladder cancer than its serum equivalent [235]. Moreover, urinary CA19-9 levels could be a new effective diagnostic tool for bladder cancer patients with both Le and Se alleles. Particularly, 70% of bladder cancer patients with both Le and Se alleles presented CA19-9 levels over the cut-off value, and only 16% of patients with other urological conditions were over the cut-off [236]. Furthermore, simultaneous elevation of CA19-9 and CEA serum levels correlated with tumour invasion and grade in patients with CA19-9-expressing urothelial carcinomas [53]. In addition, SLea antigen has been observed in bladder dysplasia, Tis, non-invasive, and invasive carcinomas of the bladder [53, 236, 237], suggesting that these patients may present elevated CA19-9, which warrants confirmation in broad clinical studies.

Considering the cancer-associated glycoproteins, galectins also hold potential for bladder cancer detection. In fact, bladder cancer patient’s serum levels of galectin-3 are considerably higher than control groups, and are correlated with tumour type, stage and grade [98, 100, 103]. Particularly, patients with high-grade urothelial carcinoma have higher serum levels of galectin-3 than those with low-grade tumours [103]. Moreover, patients with muscle-invasive tumours also have higher serum levels of galectin-3 than those with Ta tumours [103], conferring to this glycoprotein a diagnostic and stratification value for bladder cancer patients. In addition, serum levels of galectin-3 also have diagnostic value for bladder squamous cell carcinoma (SCC) patients [103]. More recently, a multiplexed immunosensor has been developed for the detection of specific biomarkers galectin-1 and lactate dehydrogenase B present in different grades of bladder cancer cell lysates. This approach has allowed not only the identification of different grades of bladder cancer cells but also the real time detection of multiple analytes on a single chip, providing more practical benefits for clinical diagnosis [238].

Regarding proteoglycans, syndecan-1 was also explored as a diagnosis biomarker in a non-invasive urine-based assay, however with low predictive value (sensitivity of 70%, specificity of 48% and accuracy of 59%) [222]. Therefore, further validation is warranted in larger and prospective studies. The chondroitin sulfate proteoglycan 6, also known as structural maintenance of chromosomes 3 (SMC3), has also been found overexpressed in bladder cancer. Moreover, when used in combination with six gene transcripts (insulin-like growth factor-binding protein 7, sorting nexin 16, cathepsin D, chromodomain helicase DNA-binding protein 2, nell-like 2, and tumour necrosis factor receptor superfamily member 7), it could discriminate bladder cancer from control blood samples with a sensitivity of 83% (95% confidence interval, 67-93%) and a specificity of 93% (95% confidence interval, 76-99%), constituting a possible blood diagnosis biomarker [239]. Beyond the prognosis role of endocan, recently this proteoglycan also demonstrated non-invasive diagnosis potential for bladder cancer. In fact, serum expression of endocan could discriminated bladder cancer patients with a sensitivity of 50% and specificity of 77% while the urinary endocan expression resulted in a sensitivity of 62% and specificity of 71% [240]. Moreover, HA and its related enzymes may have potential for detecting bladder cancer patients. In fact, increased HA levels are detected in all bladder cancer grades, while HAse levels are preferentially elevated in urothelial bladder cancer grades 2 and 3 [241, 242]. Lokeshwar *et al.* and Passerotti *et al.* have reported an optimal sensitivity (91.9% and 81.9%, respectively) and specificity (92.8% and 80.5%, respectively) of the HA test for bladder cancer detection [243, 244]. Urinary HAse measurement demonstrated a 100% sensitivity and 88.8% specificity to detect grade 2 and 3 bladder tumours [242]. Measurements of both urinary HA and HAse (the HA-HAse test) demonstrated an 89% sensitivity and 83% specificity for detecting urothelial bladder cancer [241]. Similarly, other study evaluated this non-invasive method in urine samples, showing that the HA test has 83.1% sensitivity, 90.1% specificity and 86.5% accuracy to detect bladder cancer [245]. Also, the urinary HAse test demonstrated 81.5% sensitivity, 83.8% specificity and 82.9% accuracy in detecting grade 2 and 3 bladder cancer [245]. Other studies also reported high sensitivity and specificity for detecting bladder cancer using both urinary HA and HAse by a non-invasive approach [246-249]. In fact, the sensitivity of HA-HAse test demonstrated to be superior to ImmunoCyt^®^ and cytology (83.3% *versus* 63.3% and 73.0%, respectively), as well as to BTA STAT^®^ test (94.0% *versus* 61.0%). In turn, specificity was comparable between HA-HAse and ImmunoCyt^®^ or cytology (78.1% *versus* 75.0% and 79.7%, respectively) and between HA-HAse and BTA STAT^®^ test (63.0% *versus* 74.0%) for detecting bladder cancer or bladder cancer recurrence [248, 249]. The evaluation of HAS1 and HA expression resulted in a 79% and 88% sensitivity, as well an 83.3% and 100% specificity, respectively, for detecting bladder cancer. Moreover, both expressions correlated with a positive HA urine test [247]. In addition, the combined expression of HAS2-HYAL1 detected bladder cancer with overall sensitivity of 85.4% and 79.5% specificity, predicting recurrence within 6 months [179] (Figure 6).

Despite significant research efforts and promising upfront results, neither glycans nor glycoconjugates have yet been approved for non-invasive bladder cancer detection. Again, reduced study dimensions, biased patient series and variations in detection methods are amongst the factors hampering the generalization of these approaches. As such, comprehensive clinical studies on the glycome, glycoproteome and glycolipidome of bodily fluids are warranted to broaden our understanding about alterations accompanying malignant transformations, disease progression and dissemination. Moreover, efforts should be undertaken to incorporate glycans in broad biomarker panels envisaging highly sensitive and specific detection methods.

## GLYCOMICS AND GLYCOPROTEOMICS: INSIGHTS TOWARDS PRECISION MEDICINE

The establishment of clinically useful glycan-based/assisted molecular models have been significantly delayed by serious analytical limitations. In fact, the majority of the studies presented to this date are target-directed and based on immune assays with antibodies and/or lectins whose specificity for the target ligands has not been fully disclosed yet. These aspects delay the generalization of glycans biomarker potential. Moreover, most studies are one-dimensional, failing to provide a comprehensive overview of the glycome in clinical settings. The low abundance of relevant glycoforms in biological milieus also poses a major analytical difficulty, which adds to the significant structural diversity and complexity presented by this class of biomolecules.

In the past decade, mass spectrometry has emerged as a core analytical technology to interrogate the glycome due to the rapid advance in resolution, mass accuracy, sensitivity, and reproducibility provided by modern hybrid mass analyzers. Currently, glycans and glycoconjugates analysis relies on hyphenated techniques comprehending separations by liquid chromatography or capillary electrophoresis, as well as detection by electrospray tandem mass spectrometry (ESI-MS) and matrix-assisted laser desorption/ionization time-of-flight mass spectrometry (MALDI-MS). Complementary tandem experiments are required for unambiguous assignments. Even though, the identification of isomeric and/or isobaric species remains a challenging task. Nevertheless, it has been demonstrated that certain isomeric glycans produce characteristic product ion spectra that can be used for identification, irrespectively of the type of mass spectrometer [250, 251]. Moreover, the introduction of graphitized LC columns has significantly improved the analysis of native glycans by mass spectrometry [252]. More in depth insights on analytical advances in glycomics may be found in recent reviews on the subject [253-255]. Recent developments in matrix-assisted laser desorption/ionization (MALDI) mass spectrometry imaging (MSI) on formalin-fixed paraffin-embedded (FFPE) tissue sections may also provid a key tool for evaluating spatio-temporal investigation of glycosylation changes in cancer tissues. [269]. However, while the field still struggles with technical difficulties associated with variations in accuracy depending on analytical method and variety of mass spectrometry architectures, significant efforts are ongoing to standardize protocols and implement robust glycoanalytical platforms [256, 257]. In addition, rapid expansion of high-throughput mass spectrometry studies has generated significant amounts of experimental “omics” data that require more sophisticated bioinformatics tools and databases. In this context, several groups have started the development of algorithms for computerized annotation of mass spectra and fragmentation data, as revised by Hu *et al.* [258]. However, while significant advances have been observed for glycan analysis, glycopeptide data interpretation remains immature compared to proteomics data analysis. This is partly due to lack of consensus regarding the best way of estimate the false discovery rate, and the existence of multiple formats of data storage. Nevertheless, guidelines for reporting mass spectrometry-based glycoanalytic data are being developed [257, 259].

Despite the enormous potential of glycomics, few quantitative and comprehensive studies were conducted on bladder cancer. Yang *et al.* quantitatively analyzed and compared glycan expression patterns in normal and bladder cancer cells through an integrated methodology using lectin microarray and mass spectrometry [58]. It has been demonstrated that SLe^x^ and high mannose-type *N*-glycans were highly expressed in bladder cancer cells [58]. In addition, a high expression of core-fucosylated *N*-glycans but a low expression of terminally fucosylated *N*-glycans was observed in bladder cancer cells [58]. In turn, Pocheć *et al.* determined the *N*-glycan patterns of integrin α3β1 in bladder cancer cells compared to normal bladder cells using an integrated methodology of lectin-binding assays and mass spectrometry [260]. Accordingly, bladder cancer cell-associated integrins have been found to express high-mannose, hybrid and predominantly complex type *N*-oligosaccharides, as well as the sialylated tetra-antennary complex type glycan Hex_7_HexNAc_6_FucSia_4_ [260]. Weather by focusing on the whole proteome or in a single glycoprotein, both studies have given an important *in vitro* view of the glycome pattern of bladder tumour cells compared to a normal state, paving the way for new and more comprehensive studies.

In summary, early glycan-based clinical studies have created the molecular basis to drive the emerging area of advanced glycomics in bladder cancer. We expect that the generalization of these approaches leads to the discovery of key glycan biomarkers with clinical and therapeutic potential in the near future. The incorporation of glycogenomics and glycoinformatics datasets are expected to accelerate a comprehensive understanding of the glycome. Furthermore, the integration with other “omics” will be crucial to deepen the understanding of glycosylation’s role in human systems and provide models capable of disclosing the polymorphic nature of disease and ultimately help tailoring medical decisions and achieve precision medicine settings.

Concluding remarks and future perspectives

Bladder cancer is a heterogeneous disease encompassing distinct biological features and clinical outcomes. This is responsible for elevated recurrence rates, often accompanied by disease progression facing existing treatments. Moreover, it has hampered the establishment of precision medicine settings capable of molecular-based individualization of disease management. These aspects make bladder cancer one of the costliest malignancies to manage, constituting a burden to both patients and healthcare systems. We believe that true advances in this field will require an integrative panomics approach capable of providing robust models for molecular-based patient tailored clinical decisions. So far, most efforts have been put in genomics, transcriptomics and proteomics fields, with upfront enthusiastic results; however, over 40 years of glycobiology research has yet to retrieve solid evidences capable of boosting clinical implementation. Even though many studies have highlighted glycans and glyconjugates (glycoproteins, glycolipids and proteoglycans) holding true clinical potential, few have engaged in a comprehensive interrogation of the glycome. Notwithstanding, the relevance of these molecular entities for disease progression and dissemination suggests potential for more in depth targeted omics studies. Moreover, the standardization of glycomics protocols backed by high-throughput analytical and novel bioinformatics tools opens now a unique opportunity for real advances in this area. Therefore, the field must now focus on large scale multicentric translational studies integrating glycomics data with novel molecular findings, including recently proposed models for bladder cancer risk-stratification [261-263]. Moreover, our understanding on the glycobiology of chemoresistance, formation of the pre-metastatic and metastatic niches is still scarce and warrant careful evaluation on the near future. Complementary understanding of glycan biosynthesis pathways and the biological significance of these alterations will be warranted, envisaging theragnostic applications. Taking into consideration the glycome’s strong dependence on the microenvironment and physiological status, significant efforts should put on developing models capable of recreating tumour glycoheterogeneity. Patient derived xenografts have been proven useful in this context [264] and may constitute valuable tools for understanding the dynamics of glycosylation in malignancy as well as for the identification of prognostic glycobiomarkers. Ultimately, this will be of key importance for developing targeted therapeutics while exploring the cell-surface nature of glycans. Such approaches would strongly benefit from the identification of glycoconjugates (proteins, peptides or lipids) yielding cancer-associated carbohydrate antigens, which would significantly narrow biomarker specificity for malignant cells. While the development of novel high-affinity glycan ligands, namely humanized monoclonal antibodies and antibody fragments, for theragnostics applications still poses a major task due to the complex structural nature of these molecules, several advances in cell glycoengeneiring and glycosynthesis [265-267] hold potential to overcome these limitations. Finally, it has been highlighted that glycans play a key role in immune modulation, especially by favoring tumour tolerogenic mechanisms [21, 268]. A deeper understanding of influence of glycosylation in immunological mechanisms is a hot research topic that will pave the way for circumventing these events and for more effective and less toxic immunotherapies. In summary, an intervention roadmap has been established to boost glycobiology towards omics settings capable of generating key data to improve the management of bladder cancer patients.

## SUPPLEMENTARY MATERIALS TABLE



## Abbreviations

BCG: bacillus Calmette-Guérin
CSC: cancer stem cell
ECM: extracellular matrix
EGFR: epidermal growth factor receptor
EMT: epithelial-to-mesenchymal transition
ER: endoplasmic reticulum
FDA: Food and Drug Administration
FUT-I: alpha1,2-fucosyltransferase 1
FUT-VI: alpha1,3-fucosyltransferase VI
GA: Golgi apparatus
GalNAc: N-acetylgalactosamine
GlcNAc: N-acetylglucosamine
GPAA1: GPI anchor attachment 1
HAS: hyaluronic acid synthases
IGF-IR: insulin-like growth factor receptor I
ITGA6: alpha6beta4 integrin
ITGAV: αv integrin receptors
MIBC: muscle invasive bladder cancer
MUC1: mucin 1
MVAC: methotrexate, vinblastine, cisplatin and doxorubicin
NMIBC: non-muscle invasive bladder cancer
PD-L1: programmed death-ligand 1
PGAP1: post-GPI attachment to proteins 1
ppGalNAc-Ts: UDP-GalNAc:polypeptide N-acetylgalactosaminyl transferases
NMP22: nuclear matrix protein
OSTase: oligosaccharide transferase complex
RHAMM: hyaluronan-mediated motility
RhoGDI2: RhoGTP dissociation inhibitor 2
SLea: sialyl lewisa
SLex: sialyl lewisx
STn: sialyl-Tn
TGF: transforming growth factor
TURBT: transurethral resection of the bladder tumour
