# Supplementary material for: Over forty years of bladder cancer glycobiology: Where do glycans stand facing precision oncology?
Source: Oncotarget. 2017 Jul 21;8(53):91734–64. doi: 10.18632/oncotarget.19433 (PMC5710962; doi:10.18632/oncotarget.19433)
Supplement: Supplementary file 1 [file oncotarget-08-91734-s001.docx]

Over forty years of bladder cancer glycobiology: Where do glycans stand facing precision oncology?

**SUPPLEMENTRAY MATERIALS**

| **Table S1. Biological and clinical significance of altered glycans** **and related biosynthetic enzymes in bladder cancer** | | | | | | | |
| --- | --- | --- | --- | --- | --- | --- | --- |
|  | **Type of sample (n)** | **Biological samples** | **Main techniques** | **% positive in bladder tumours** | **% positive in normal bladder epithelium** | **Biological/Clinical significance** | **Refs** |
| **Protein glycosylation** | | | | | | | |
| ***Cancer-associated alterations in N-glycosylation*** | | | | | | | |
| **GnT-III** | Normal bladder epithelium (n=10)  Urothelial bladder cancer (n=10)  - *Grade:* pG1 (n=3), pG2 (n=4), pG3 (n=3)  - *Stage:* pT1 (n=5), pT2 (n=4), pT3 (n=1) | Tissue | HPLC | Not Specified | Not specified | Overexpression associated with higher stage and grade | [[30](#_ENREF_30)] |
| **Bis-GlcNAc antennae** | Normal bladder epithelium (n=10)  Urothelial bladder cancer (n=10)  - *Grade:* pG1 (n=3), pG2 (n=4), pG3 (n=3)  - *Stage:* pT1 (n=5), pT2 (n=4), pT3 (n=1) | Tissue | HPLC | Not Specified | Not specified | Overexpression associated with higher stage and grade | [[30](#_ENREF_30)] |
| **GnT-IV** | Normal bladder epithelium (n=10)  Urothelial bladder cancer (n=10)  - *Grade:* pG1 (n=3), pG2 (n=4), pG3 (n=3)  - *Stage:* pT1 (n=5), pT2 (n=4), pT3 (n=1) | Tissue | HPLC | Not Specified | Not specified | Overexpression associated with higher stage | [[30](#_ENREF_30)] |
| **GnT-V** | NMIBC (n=60):  - *Grade:* G1 (n=9), G2 (n=43), G3 (n=8)  - *Stage*: pTa (n=44), pT1 (n=16) | FFPE | IHC | NMIBC (52%)  - *Grade:* G1 (78%), G2 (49%), G3 (38%)  - *Stage*: pTa (60%), pT1 (31%) | Not specified | Reduction of expression associated with higher grade and stage, shorter disease-free survival and RFS | [[31](#_ENREF_31)] |
|  | Normal bladder epithelium (n=15)  Dysplasia (n=3)  NMIBC and MIBC (n=164):  - *Grade:* G1 (n=19), G2 (n=74), G3 (n=71)  - *Stage:* pTa (n=62), pT1 (n=37), pT2 (n=33), pT3/4 (n=32) | FFPE | IHC | Dysplasia (100%)  NMIBC and MIBC (40%)  - *Grade:* G1 (79%), G2 (47%), G3 (22%)  - Stage: Ta (68%), T1 (32%), T2 (27%), T3/4 (13%) | 0% | Reduction of expression associated with higher grade, invasion and shorter cause-specific survival | [[32](#_ENREF_32)] |
|  | Normal bladder epithelium (n=10)  Urothelial bladder cancer (n=10)  - *Grade:* pG1 (n=3), pG2 (n=4), pG3 (n=3)  - *Stage:* pT1 (n=5), pT2 (n=4), pT3 (n=1) | Tissue | HPLC | Not Specified | Not specified | NS for stage or grade | [[30](#_ENREF_30)] |
| **β1,6-GlcNAc antennae** | Normal bladder epithelium (n=4)  NMIBC pTa (n=2) and MIBC pT2/3 (n=4) | Tissue | HPLC | Not Specified | Not specified | Overexpression associated with lower stage | [[32](#_ENREF_32)] |
| **ABH(O) antigens** | Urothelial bladder cancer (n=81)  - *Grade:* pG1 (n=7), pG2 (n=23), pG3 (n=51)  - *Stage:* pTa (n=14), pT1 (n=36), pT2 (n=12), pT3 (n=10), pT4 (n=9) | FFPE | IHC | Urothelial bladder cancer (31%)  - A (35%) | 100% | NS for stage or grade | [[39](#_ENREF_39)] |
|  | Normal bladder epithelium (n=35)  Urothelial bladder cancer (n=48) | Blood samples  Frozen tissues | Modified RCA test | Urothelial bladder cancer (56%)  - O (69%)  - A (40%)  - B (80%)  - AB (100%) | 100% | Loss/ reduction associated with higher grade and invasion | [[44](#_ENREF_44)] |
|  | Urothelial bladder cancer (n=39)  - *Grade:* G1 (26%), G2 (56%), G3 (18%) | FFPE | IHC | 46% | Not specified | Loss/ reduction associated with invasion | [[45](#_ENREF_45)] |
|  | Urothelial bladder cancer (n=19) | FFPE | IHC | Urothelial bladder cancer (74%)  - O (63%)  - A (50%)  - B (0%)  - AB (50%) | 100% | Loss/ reduction associated with invasion | [[46](#_ENREF_46)] |
|  | Normal bladder epithelium (n=21)  Urothelial bladder cancer (n=5)  - *Grade:* G2 (80%), G3 (20%)  - *Stage:* T1 (20%), T2 (60%), T3 (20%) | Frozen tissues | IF | Urothelial bladder cancer (0%)  - O (0%)  - A (0%) | 100% | Loss/ reduction associated with progression and shorter RFS | [[47](#_ENREF_47)] |
| **Lewis^a^ + Lewis^b^** | Normal bladder epithelium (n=35)  Urothelial bladder cancer (n=48) | Blood samples  Frozen tissues | Modified RCA test | Urothelial bladder cancer (48%)  - Le^a-b+^ (48%)  - Le^a+b-^ (64%)  - Le^a-b-^ (0%) | Normal bladder epithelium (74%)  - Le^a-b+^ (67%)  - Le^a+b-^ (91%) | Loss/ reduction associated with higher grade and invasion | [[44](#_ENREF_44)] |
|  | Urothelial bladder cancer (n=19) | Saliva samples  Blood samples  FFPE | EIA  IHC | - Le^a-b+^ (16%)  - Le^a+b+^ (53%)  - Le^a-b-^ (31%) | - Le^a-b+^ (16%)  - Le^a+b-^ (5%)  - Le^a+b+^ (58%)  - Le^a-b-^ (21%) | NS for invasion | [[46](#_ENREF_46)] |
|  | Urothelial bladder cancer (n=93)  - *Grade:* G2 (62%), G3 (38%)  - *Stage:* pTa (71%), pT1-pT3 (29%) | Blood samples  FFPE | IHC | Urothelial bladder cancer (71%)  - Le^a+b-^ and Le^a-b+^ (73%)  - Le^a-b-^ (50%) | Not specified | Loss/ reduction associated with higher grade and invasion and shorter RFS | [[49](#_ENREF_49)] |
| **Lewis^x^ (SSEA-1)** | Urothelial bladder cancer (n=19) | Saliva samples  Blood samples  FFPE | EIA  IHC | 100% | 0% (expect occasional umbrella cells) | Expression associated with invasion | [[46](#_ENREF_46)] |
|  | Urothelial bladder cancer (n=26)  - *Grade:* G1 (23%), G2 (31%), G3 (46%)  - *Stage:* pTa (42%), pT1 (19%), pT2 (8%), pT3 (27%), T4 (4%)  Renal pelvis and ureter cancer (n=26) | FFPE | IHC | 98% (for both urothelial bladder cancer and renal pelvis and ureter cancer) | Not specified | NS for grade | [[50](#_ENREF_50)] |
| **Lewis**^y^ | Urothelial bladder cancer (n=19) | Saliva samples  Blood samples  FFPE | EIA  IHC | 100% | 68% | Expression associated with invasion | [[46](#_ENREF_46)] |
| **Sialyl Lewis^a^**  **(CA19-9)** | Urothelial bladder cancer (n=26)  - *Grade:* G1 (23%), G2 (31%), G3 (46%)  - *Stage:* pTa (42%), pT1 (19%), pT2 (8%), pT3 (27%), pT4 (4%)  Renal pelvis and ureter cancer (n=26) | FFPE | IHC | 19% (for both urothelial bladder cancer and renal pelvis and ureter cancer) | Not specified | Loss/reduction associated with higher malignant potential | [[50](#_ENREF_50)] |
|  | Normal bladder epithelium (n=25)  Urothelial bladder cancer (n=75)  - *Grade:* Low (55%), High (45%)  - *Stage:* Ta (16%), T1 (52%), T2 (16%), T3 (7%), T4 (9%) | Blood samples | EIA | 37% | Not specified | Diagnosis – expressed in blood | [[234](#_ENREF_234)] |
|  | Normal bladder epithelium (n=50)  Urothelial bladder cancer (n=55)  - *Grade:* Low (64%), High (36%) | Blood samples  Urine samples | EIA | Elevated compared to normal bladder epithelium (not specified) | Not specified | Diagnosis – expressed in blood and urine | [[235](#_ENREF_235)] |
|  | Dysplasia (n=2)  Bladder cancer (n=119)  - *Grade:* G0 (3%), G1 (43%), G2 (42%), G3 (12%)  - *Stage:* Tis (3%), Ta (54%), T1 (31%), T2 (6%), T3 (4%), T4 (2%)  - Adenocarcinoma (n=1)  - Not determined (n=6)  Other urologic diseases (n=31) | Urine samples  Blood samples | EIA  PCR | 70% | Not specified in normal bladder epithelium  16% of patients with other urologic diseases | Diagnosis – expressed in urine | [[236](#_ENREF_236)] |
|  | Normal bladder epithelium (n=71)  Urothelial bladder cancer (n=146)  - *Grade:* G1 (28%), G2 (47%), G3 (25%)  - *Stage:* pTa (48%), pT1 (26%), pT2 (13%), pT3/pT4/N+ (13%) | Blood samples  Tissues | EIA  IHC | Not Specified | Not specified | Serum overexpression associated with higher stage, grade and invasion | [[53](#_ENREF_53)] |
|  | Normal bladder epithelium (n=50)  Urothelial bladder cancer (n=47) | Blood samples  Urine samples | EIA | Elevated compared to normal bladder epithelium (not specified) | Not specified | Diagnosis – expressed in blood and urine | [[237](#_ENREF_237)] |
| **Sialyl Lewis^x^** | Urothelial bladder cancer (n=44)  - *Grade:* G2 (34%), G3 (66%)  - *Stage:* pT2 (55%), pT3 (43%), pT4 (2%) | FFPE | IHC | 70% | Not specified | Expression associated with invasion and shorter overall survival | [[54](#_ENREF_54)] |
|  | Urothelial bladder cancer (n=26)  - *Grade:* G1 (23%), G2 (31%), G3 (46%)  - *Stage:* pTa (42%), pT1 (19%), pT2 (8%), pT3 (27%), T4 (4%)  Renal pelvis and ureter cancer (n=26) | FFPE | IHC | 100% (for both urothelial bladder cancer and renal pelvis and ureter cancer) | Not specified | NS for grade | [[50](#_ENREF_50)] |
| ***Cancer-associated alterations in O-glycosylation*** | | | | | | | |
| **Tn-antigen** | Normal bladder epithelium (n=10)  Bladder cancer (n=34)  - *Stage:* Initially all were Ta | FFPE | IHC | 79% | 0% | Loss/reduction associated with higher stage | [[67](#_ENREF_67)] |
| **Sialyl-Tn antigen** | Normal bladder epithelium (n=6)  Bladder cancer (n=69)  - *Stage:* NMIBC (72%), MIBC (28%) | FFPE | IHC | 52% | 0% | Overexpression associated with higher stage and invasion | [[64](#_ENREF_64)] |
|  | Normal bladder epithelium (n=10)  Bladder cancer (n=34)  - *Stage:* Initially all were Ta | FFPE | IHC | 3% | 10% | NS for invasion | [[67](#_ENREF_67)] |
|  | Urothelial bladder cancer (n=96)  - *Grade:* Low (17%), High (83%)  - *Stage:* Ta (28%), T1 (21%), T2 (9%), T3 (21%), T4 (21%) | FFPE | IHC | 60% | Not specified | Overexpression associated with higher stage and shorter cancer-specific survival | [[20](#_ENREF_20)] |
|  | Urothelial bladder cancer (n=96)  - *Grade:* Low (40%), High (60%)  - *Stage:* Ta (43%), T1 (57%) | FFPE | IHC | 66% | Not specified | Expression associated with BCG response and higher RFS | [[75](#_ENREF_75)] |
| **ST6GalNAc.I** | Bladder cancer (n=69)  - *Stage:* NMIBC (72%), MIBC (28%) | FFPE | qPCR | 53% in MIBC | Not specified | Overexpression associated with higher stage and sialyl-Tn levels | [[64](#_ENREF_64)] |
| **T-antigen** | Urothelial bladder cancer (n=39)  - *Grade:* G1 (26%), G2 (56%), G3 (18%) | FFPE | IHC | 38% | Not specified | NS for invasion or RFS | [[45](#_ENREF_45)] |
|  | Bladder cancer (n=73) | Blood samples  FFPE | EIA  IF | 55% | 0% | Expression associated with higher grade and shorter RFS | [[66](#_ENREF_66)] |
|  | Normal bladder epithelium (n=10)  Bladder cancer (n=34)  - *Stage:* Initially all were Ta | FFPE | IHC | 29% | 0% | Expression associated with invasion | [[67](#_ENREF_67)] |
|  | Urothelial bladder cancer (n=96)  - *Grade:* Low (17%), High (83%)  - *Stage:* Ta (28%), T1 (21%), T2 (9%), T3 (21%), T4 (21%) | FFPE | IHC | 10% | Not specified | NS for stage or cancer-specific survival | [[20](#_ENREF_20)] |
|  | Urothelial bladder cancer (n=56)  - *Stage:* NMIBC (59%), MIBC (41%) | FFPE  Frozen tissues | IHC  Modified RCA test | 65% MIBC  10% NMIBC | 0% | Expression associated with higher stage | [[76](#_ENREF_76)] |
| **s6T** | Urothelial bladder cancer (n=96)  - *Grade:* Low (40%), High (60%)  - *Stage:* Ta (43%), T1 (57%) | FFPE | IHC | 31% | Not specified | Combined expression with STn associated with BCG response and higher RFS | [[75](#_ENREF_75)] |
| **ST3Gal.I** | Normal bladder epithelium (n=4)  Bladder cancer (n=49)  - *Stage:* NMIBC (88%), MIBC (12%) | Tissues | qPCR | Not specified | Not specified | Overexpression associated with higher stage and shorter RFS | [[65](#_ENREF_65)] |
| ***Overexpression of cancer-associated membrane glycoproteins*** | | | | | | | |
| **HER2** | Nine studies with 2,242 eligible bladder cancer patients (meta-analysis) | Tissues | IHC | 41% | Not specified | Overexpression associated with higher grade, metastasis, and shorter disease-specific survival and disease-free survival | [[82](#_ENREF_82)] |
| **EpCAM** | Normal bladder epithelium (n=53)  Urothelial bladder cancer (n=607)  - *Grade:* G1 (22%), G2 (30%), G3 (48%)  - *Stage:* Ta (49%), T1 (28%), T2+ (23%) | Urine samples | EIA | Elevated compared to normal bladder epithelium (not specified) | Not specified | Overexpression associated with higher grade, stage and shorter cancer-specific survival | [[86](#_ENREF_86)] |
|  | Urothelial bladder cancer (n=99) | Tissues | IHC | Not specified | Not specified | Overexpression associated with higher grade, stage and shorter overall survival | [[87](#_ENREF_87)] |
| **Galectin-1** | Urothelial bladder cancer (n=185)  - *Grade:* Low (18%), High (82%)  - *Stage:* Ta (31%), T1 (35%), T2 (22%), T3 (6%), T4 (6%) | Frozen tissues | IHC  qPCR | 30% | Not specified | Overexpression associated with invasion and shorter disease-specific survival | [[96](#_ENREF_96)] |
|  | Normal bladder epithelium (n=5)  Urothelial bladder cancer (n=38)  - *Grade:* G0 (8%), G1 (40%), G2 (26%), G3 (26%)  - *Stage:* Ta (50%), T1 (18%), T2 (11%), T3 (21%) | FFPE | Northern, Southern and  Western blotting  IHC | 34% for patients with grade>0 | Very low levels (not specified) | Overexpression associated with higher grade and stage | [[97](#_ENREF_97)] |
| **Galectin-3** | Normal bladder epithelium (n=5)  Urothelial bladder cancer (n=38)  - *Grade:* G0 (8%), G1 (40%), G2 (26%), G3 (26%)  - *Stage:* Ta (50%), T1 (18%), T2 (11%), T3 (21%) | FFPE | Northern and Southern  blotting | 58% for patients with grade>0 | Very low levels (not specified) | NS for stage and grade | [[97](#_ENREF_97)] |
|  | Normal bladder epithelium (n=24)  Bladder cancer (n=43)  - *Grade:* G1 (5%), G2 (49%), G3 (46%)  - *Stage:* NMIBC (77%), MIBC (23%) | Blood samples | EIA | Not specified | Not specified | Diagnosis – overexpressed in blood | [[100](#_ENREF_100)] |
|  | Urothelial bladder cancer (n=494)  - *Grade:* G1 (7%), G2 (19%), G3 (74%)  - *Stage:* pTa (1%), pT1 (44%), pT2+ (55%) | Frozen tissues  FFPE  Urine samples | Arrays  IHC  EIA | Not specified | Not specified | Overexpression associated with higher stage and grade, and shorter overall survival (T1G3 tumours) | [[101](#_ENREF_101)] |
|  | Normal bladder epithelium (n=10)  Urothelial bladder cancer (n=35)  - *Grade:* Low (49%), High (51%)  - *Stage:* NMIBC (49%), MIBC (51%)  Squamous cell carcinoma (n=10)  Cystitis (n=15) | Tissues  Blood samples | IHC  EIA | Elevated in bladder cancer compared to the other groups (not specified) | Week (not specified) | Overexpression associated with higher grade  Diagnosis – overexpressed in blood | [[102](#_ENREF_102)] |
|  | Normal bladder epithelium (n=10)  Urothelial bladder cancer (n=35)  Squamous cell carcinoma (n=10) | Blood samples | EIA | Not specified | Not specified | Overexpression associated higher stage and stage  Diagnosis – overexpressed in blood | [[103](#_ENREF_103)] |
| **Galectin-7** | Normal bladder epithelium (n=4)  Urothelial bladder cancer (n=17)  - *Stage:* cT3 or greater (100%) | Tumour cell lines  Frozen tissues | Dose-response assay  qPCR  Western blotting | 53% | Not specified | Overexpression associated with lower stage and reduced chemoresistance | [[104](#_ENREF_104)] |
| **Galectin-8** | Normal bladder epithelium (n=10)  Urothelial bladder cancer (n=187)  - *Stage:* NMIBC (87%), MIBC (13%) | Tissues | IHC | Loss of expression | High expression (not specified) | Loss associated with higher grade and stage and shorter RFS | [[105](#_ENREF_105)] |
| **CD44** | Normal bladder epithelium (n=6)  Urothelial bladder cancer (n=30)  - *Grade:* Low (57%), High (43%)  - *Stage:* T1 (23%), T2 (47%), T3 (30%)  Squamous cell carcinomas (n=20) | FFPE | IHC | 83% | 83% | Expression associated with higher grade and stage | [[120](#_ENREF_120)] |
|  | Urothelial bladder cancer (n=173)  - *Grade:* G1 (30%), G2 (48%), G3 (22%)  - *Stage:* Ta (13%), T1 (39%), T2 (27%), T3 (15%), T4 (6%) | Tissues | IHC | 2% | Not specified | Expression associated with higher grade, stage, mitotic index, density of tumour infiltrating lymphocytes and shorter overall survival | [[121](#_ENREF_121)] |
|  | pTa/pT1 grade 2 and 3 bladder cancer (n=66) | FFPE | IHC | Loss of expression | Not specified | Expression associated with higher progression-free survival | [[124](#_ENREF_124)] |
| **CD44v6** | Normal bladder epithelium (n=6)  Urothelial bladder cancer (n=30)  - *Grade:* Low (57%), High (43%)  - *Stage:* T1 (23%), T2 (47%), T3 (30%)  Squamous cell carcinomas (n=20) | FFPE | IHC | 77% | 67% | Expression associated with higher grade and stage | [[120](#_ENREF_120)] |
|  | Urothelial bladder cancer (n=173)  - *Grade:* G1 (30%), G2 (48%), G3 (22%)  - *Stage:* Ta (13%), T1 (39%), T2 (27%), T3 (15%), T4 (6%) | Tissues | IHC | 84% | Not specified | Expression associated with lower grade, stage, mitotic index and higher overall survival | [[121](#_ENREF_121)] |
|  | Urothelial bladder cancer (n=410)  - *Grade:* G1 (10%), G2 (30%), G3 (51%), CIS (8%)  - *Stage:* Tis (8%), Ta (30%), T1 (19%), T2 (24%), T3 (15%), T4 (4%) | FFPE | IHC | 81% | Not specified | Expression associated with lower grade and stage and higher RFS and overall survival | [[122](#_ENREF_122)] |
| **CD44v9** | pT1 high-grade bladder cancer (n=98) | FFPE | IHC | Not specified | Not specified | Overexpression associated with shorter progression-free and cancer-specific survival | [[125](#_ENREF_125)] |
| **MUC1** | Urothelial bladder cancer (n=539) | FFPE | IHC | 62% | Not specified | Expression associated with higher grade | [[129](#_ENREF_129)] |
|  | Urothelial bladder cancer (n=82) | Tissues | qPCR | Not specified | Not specified | Overexpression associated higher overall survival only when there is co-overexpression of HER3 | [[131](#_ENREF_131)] |
| **MUC2** | Urothelial bladder cancer (n=539) | FFPE | IHC | 40% | Not specified | Expression associated with lower grade and stage, reduced cancer-specific death and higher overall survival | [[129](#_ENREF_129)] |
| **MUC4** | Urothelial bladder cancer (n=539) | FFPE | IHC | 27% | Not specified | Expression associated with higher cancer-specific death | [[129](#_ENREF_129)] |
| **MUC6** | Urothelial bladder cancer (n=539) | FFPE | IHC | 22% | Not specified | Expression associated with lower grade and stage, reduced cancer-specific death and higher overall survival | [[129](#_ENREF_129)] |
| **ITGA6** | Urothelial bladder cancer (n=57) | Tissues | IHC | 37% | Not specified | Overexpression associated with shorter overall survival | [[133](#_ENREF_133)] |
| **ITGAV** | Normal bladder epithelium (n=34)  Urothelial bladder cancer (n=61)  - *Stage:* pTa (21%), pTis (10%), pT1 (13%), pT2 (11%), pT3 (28%), T4 (17%) | FFPE  Frozen tissues | IHC  qPCR | 46% | 13% | Overexpression associated with higher stage and grade | [[139](#_ENREF_139)] |
| ***Proteoglycan glycosylation*** | | | | | | | |
| ***Alterations in cancer-associated transmembrane proteoglycans*** | | | | | | | |
| **Syndecan-1** | Normal bladder epithelium (n=15)  Bladder cancer (n=198)  - *Grade:* G1 (10%), G2 (22%), G3 (55%)  - *Stage:* Ta (16%), T1 (22%), T2 (21%), T3 (28%), T4 (13%) | FFPE  Blood samples | IHC  EIA | 63% in serum  83% in membrane  40% in stroma | 100% in membrane  Serum levels similar to bladder cancer | Serum overexpression associated with higher stage and grade and invasion  Membranous loss/reduction of expression associated with higher grade  Stromal overexpression related with higher grade and stage and shorter overall survival | [[158](#_ENREF_158)] |
|  | Normal bladder epithelium (n=206+8)  Bladder cancer (n=102+185)  - *Grade:* Low (37%+15%), High (63%+85%)  - *Stage:* Tis (6%+9%), Ta (40%+24%), T1 (14%+34%), ≥T2 (40%+33%) | Urine samples + FFPE | EIA + IHC | 52% in membrane  55% in cytoplasm in high-grade tumours | 70% in membrane  Urinary levels similar to bladder cancer | Loss of membranous expression associated with higher stage and grade  Cytoplasmic overexpression associated with higher stage  Urinary overexpression associated with lower grade and stage | [[159](#_ENREF_159)] |
|  | Bladder cancer (n=109)  - *Grade:* G1 (17%), G2 (53%), G3 (30%)  - *Stage:* Ta (48%), T1 (52%) | FFPE | IHC | 63% | Not specified | Loss of expression associated with higher stage and grade and shorter RFS | [[160](#_ENREF_160)] |
|  | Urothelial bladder cancer (n=51)  - *Grade:* Low (41%), High (59%)  - *Stage:* pTis (14%), pTa (41%), pT1 (18%), ≥pT2 (27%) | FFPE | IHC | 74% at initial diagnosis of pTa⁄pT1 | Not specified | Loss of expression associated with lower grade and non-invasive tumours and shorter RFS | [[161](#_ENREF_161)] |
|  | Normal bladder epithelium (n=9)  Primary Ta bladder cancer (n=25) | FFPE  Frozen tissues | IHC  Microarray | 99% | Not specified | Cytoplasmic overexpression associated with Ta tumours | [[162](#_ENREF_162)] |
|  | Normal bladder epithelium (n=63)  Urothelial bladder cancer (n=64)  - *Grade:* Low (14%), High (86%)  - *Stage:* Tis (9%), Ta (23%), T1 (14%), T2 (48%), T3 (6%), T4 (3%) | Urine samples | EIA | Not specified | Not specified | NS for diagnosis | [[222](#_ENREF_222)] |
| **Neuropilin-1** | Urothelial bladder cancer (n=139)  - *Grade:* G1 (23%), G2-G3 (77%)  - *Stage:* ≤T1 (68%), T2-T4 (32%) | FFPE | IHC | 56% | 16% in adjacent non-malignant areas | Overexpression associated with higher stage and grade and shorter overall survival | [[165](#_ENREF_165)] |
| **Neuropilin-2** | Urothelial bladder cancer (n=247)  - *Grade:* G1 (1%), G2 (9%), G3 (90%)  - *Stage:* Ta (1%), T1 (32%), T2 (55%), T3 (9%), T4 (3%) | FFPE | IHC | 71% | Not specified | Overexpression associated with shorter overall survival, cancer-specific survival and higher early cancer-specific death | [[166](#_ENREF_166)] |
| **Neuropilin-2 + VEGF-C** | Urothelial bladder cancer (n=247)  - *Grade:* G1 (1%), G2 (9%), G3 (90%)  - *Stage:* Ta (1%), T1 (32%), T2 (55%), T3 (9%), T4 (3%) | FFPE | IHC | Not specified | Not specified | Overexpression associated with shorter overall survival | [[166](#_ENREF_166)] |
| ***Alterations in cancer-associated extracellular matrix proteoglycans*** | | | | | | | |
| **Versican** | Bladder cancer (n=5) | Tumour cell lines  Tissues | qPCR  TEM | Not specified | Not specified | Overexpression associated with invasion and poor survival | [[167](#_ENREF_167)] |
|  | Normal bladder epithelium (n=15)  Bladder cancer (n=46)  - *Stage:* Ta (54%), ≥pT2 (46%) | Tumour cell lines  Tissues | RMA  Microarray | Not specified | Not specified | Overexpression associated with invasion and stage | [[168](#_ENREF_168)] |
| **Chondroitin sulfate proteoglycan 6** | Normal bladder epithelium (n=27)  Bladder cancer (n=40) | Blood samples | Microarray | Not specified | Not specified | Diagnostic value (in a combination of six gene ratios) - expressed in blood | [[239](#_ENREF_239)] |
| **Decorin** | Normal bladder epithelium (n=4)  Bladder cancer (n=162) | Tumour cell lines  Frozen tissues | MIA  Microarray | Not specified | Not specified | Increased secretion associated with invasion and angiogenesis | [[171](#_ENREF_171)] |
|  | Bladder cancer (n=199)  - *Grade:* G1 (4%), G2 (36%), G3 (60%)  - *Stage:* ≤pT1 (46%), pT2 (21%), pT3 (25%), pT4 (7%) | Tissues  Tumour cell lines | IHC  qPCR | Not specified | Not specified | Diagnostic value - not expressed in tumour tissues (Tumour suppressor) | [[172](#_ENREF_172)] |
|  | Urothelial bladder cancer (n=40)  - *Grade:* Low (50%), High (50%) | FFPE | IHC | Not specified | Not specified | Diagnostic value - not expressed in tumour tissues (Tumour suppressor) | [[173](#_ENREF_173)] |
| **Biglycan** | Bladder cancer (n=199)  - *Grade:* G1 (4%), G2 (36%), G3 (60%)  - *Stage:* ≤pT1 (46%), pT2 (21%), pT3 (25%), pT4 (7%) | Tissues | IHC | Not specified | Not specified | Overexpression associated with invasion | [[172](#_ENREF_172)] |
|  | Urothelial bladder cancer (n=120)  - *Stage:* ≤pT1 (46%), pT2 (18%), pT3 (21%), pT4 (16%) | Frozen tissues  FFPE | qPCR  IHC | Not specified | Not specified | Overexpression associated with higher stages, invasion, proliferation inhibition and higher survival | [[176](#_ENREF_176)] |
| **Endocan** | Normal bladder epithelium (n=60)  Urothelial bladder cancer (n=148) | Frozen tissues  FFPE  Blood samples | IHC  EIA | 27% | 0% in blood vessels and tissues | Increased secretion associated with higher stages, invasion and shorter RFS | [[177](#_ENREF_177)] |
|  | Normal bladder epithelium (n=51)  Bladder cancer (n=50)  Urinary tract infection (n=50) | Serum samples  Urine samples | EIA | Higher than in normal bladder epithelium (not specified) | Not specified | Diagnosis – expressed in urine and serum | [[240](#_ENREF_240)] |
| **Hyaluronic acid** | Normal bladder epithelium (n=25)  Bladder cancer (n=144)  Other urologic diseases (n=45) | Urine samples | EIA | Not specified | Not specified | Diagnosis – expressed in urine | [[243](#_ENREF_243)] |
|  | Urothelial bladder cancer (n=160)  - *Grade:* Low (35%), High (65%)  - *Stage:* Tis (16%), Ta (27%), T1 (34%), T2 (17%), T3 (4%), T4 (1%) | Urine samples | EIA | Not specified | Not specified | Diagnosis – expressed in urine | [[244](#_ENREF_244)] |
|  | Bladder cancer (n=178)  - *Grade:* G1 (29%), G2 (50%), G3 (21%)  - *Stage:* Ta (46%), T1 (35%), T2 (10%), T3 (7%), T4 (2%) | FFPE | IHC | Not specified | Not specified | Detecting invasion | [[181](#_ENREF_181)] |
| **Hyaluronidase (HYAL1)** | Normal bladder epithelium (n=20)  Bladder cancer (n=71)  - *Grade:* G1 (31%), G2 (13%), G3 (56%)  Other urologic diseases (n=48) | Urine samples | EIA | 100% in grade 2 and 3 | 0% | Diagnosis of grade 2 and grade 3 – expressed in urine | [[242](#_ENREF_242)] |
|  | Normal bladder epithelium (n=148)  Bladder cancer (n=72) | Frozen tissues  FFPE  Urine samples | IHC  qPCR  EIA | Not specified | Not specified | Detecting invasion and higher probability of disease-specific mortality | [[179](#_ENREF_179)] |
|  | Bladder cancer (n=178)  - *Grade:* G1 (29%), G2 (50%), G3 (21%)  - *Stage:* Ta (46%), T1 (35%), T2 (10%), T3 (7%), T4 (2%) | FFPE | IHC | Not specified | Not specified | Detecting invasion and recurrence | [[181](#_ENREF_181)] |
| **Hyaluronic acid + Hyaluronidase (HA-HAse test)** | Normal bladder epithelium (n=46)  Urothelial bladder cancer (n=97)  - *Grade:* G1 (30%), G2 (30%), G3 (40%)  - *Stage:* Tis (21%), Ta (39%), T1 (7%), T2 (16%), T3 (17%)  Other urologic diseases (n=51) | Urine samples | EIA | 20% + 20% | 20% + 20% | Diagnosis regardless of grade + Diagnosis of grade 1 and grade 2 – expressed in urine | [[241](#_ENREF_241)] |
|  | Normal bladder epithelium or other urologic diseases (n=252)  Bladder cancer (n=270) | Urine samples | EIA | 80% + 80% of G2/G3 | 10% + 20% | Diagnosis regardless of grade + Diagnosis of grade 1 and grade 2 – expressed in urine | [[245](#_ENREF_245)] |
|  | Normal bladder epithelium (n=12)  Bladder cancer (n=71)  - *Grade:* G1 (11%), G2 (34%), G3 (55%)  - *Stage:* Tis (10%), Ta (20%), T1 (11%), T2 (14%), T3 (42%), T4 (3%) | FFPE  Urine samples | IHC  EIA | 89% + 77% | 0% + 0% | Diagnosis – expressed in urine | [[246](#_ENREF_246)] |
|  | Normal bladder epithelium (n=15)  Bladder cancer (n=33)  - *Grade:* G1 (18%), G2/G3 (82%)  - *Stage:* NMIBC (21%), MIBC (79%) | Urine samples  Frozen tissues and FFPE  Tumour cell lines | EIA  IHC  RT-PCR | Not specified | Not specified | Diagnosis – expressed in urine | [[247](#_ENREF_247)] |
|  | Normal bladder epithelium (n=64)  Urothelial bladder cancer (n=30)  - *Grade:* G1 (13%), G2 (50%), G3 (37%)  - *Stage:* pTis (7%), pTa (47%), pT1 (30%), pT2 (16%) | Urine samples | EIA | Not specified | Not specified | Diagnosis – expressed in urine | [[248](#_ENREF_248)] |
|  | Bladder cancer (n=70) | Urine samples | EIA | Not specified | Not specified | Diagnosis and detection of recurrence – expressed in urine | [[249](#_ENREF_249)] |
| **Hyaluronic acid synthase 1** | Normal bladder epithelium (n=15)  Bladder cancer (n=33)  - *Grade:* G1 (18%), G2/G3 (82%)  - *Stage:* NMIBC (21%), MIBC (79%) | Urine samples  Frozen tissues and FFPE  Tumour cell lines | EIA  IHC  RT-PCR | Not specified | Not specified | Diagnosis and detection of recurrence – expressed in urine | [[247](#_ENREF_247)] |
|  | Normal bladder epithelium (n=148)  Bladder cancer (n=72) | Frozen tissues  FFPE  Urine samples | IHC  qPCR  EIA | Not specified | Not specified | Overexpression was associated with invasion | [[179](#_ENREF_179)] |
| **Hyaluronic acid synthase 2-HYAL1** | Normal bladder epithelium (n=148)  Bladder cancer (n=72) | Frozen tissues  FFPE  Urine samples | IHC  qPCR  EIA | Not specified | Not specified | Diagnosis and detection of recurrence – expressed in urine | [[179](#_ENREF_179)] |
| **RHAMM** | Urothelial bladder cancer (n=120)  - *Grade:* G1 (17%), G2 (36%), G3 (47%)  - *Stage:* Ta (26%), T1 (20%), T2 (17%), T3 (21%), T4 (16%) | Frozen tissues  FFPE | qPCR  IHC | 25% | Not specified | Overexpression associated with higher stage, invasion, increased proliferation and shorter disease-specific and overall survival | [[180](#_ENREF_180)] |
| ***Lipid glycosylation*** | | | | | | | |
| ***Alterations in sphingolipids’ glycosylation*** | | | | | | | |
| **Glucosylceramide synthase** | Bladder cancer (n=75)  - *Stage:* ≤T1 (60%), T2 (37%), T3 (3%) | FFPE  Frozen tissues | IHC  Western blotting | 61% | Not specified | Overexpression associated with higher grade, invasion, poor survival and disease-free survival | [[198](#_ENREF_198)] |
| **GM3** | Normal bladder epithelium (n=2)  Bladder cancer (n=14)  - *Grade:* G1 (7%), G2 (64%), G3 (29%)  - *Stage:* Ta (14%), T1 (30%), T2 (21%), T3 (21%), T4 (14%) | Frozen tissues  Tumour cell lines | IHC  Enzyme assay | 100% in NMIBC | Not specified | Overexpression associated with reduced invasion potential | [[199](#_ENREF_199)] |
| **Gb3 and GD3 synthases** | Normal bladder epithelium (n=2)  Bladder cancer (n=14)  - *Grade:* G1 (7%), G2 (64%), G3 (29%)  - *Stage:* Ta (14%), T1 (30%), T2 (21%), T3 (21%), T4 (14%) | Frozen tissues  Tumour cell lines | IHC  Enzyme assay | Not specified | Not specified | Loss/reduction associated with reduced invasion potential and both GM3 and its synthase accumulation in NMIBC | [[199](#_ENREF_199)] |
| ***Alterations in glycosylphosphatidylinositol-anchored molecules*** | | | | | | | |
| **PIG-U** | Invasive, high-grade uroepithelial carcinomas (n=11)  Bladder cancer (n=9) | FFPE  Tumour cell lines | qPCR  Northern and Southern blotting | One-third of  cell lines and FFPE | Not specified | Overexpression associated with increased proliferation and upregulation of the urokinase receptor | [[208](#_ENREF_208)] |
|  | Normal bladder epithelium (n=14)  Bladder cancer (n=73)  - *Grade:* G1 (52%), G2/3 (48%)  - *Stage:* Ta (44%), T1 (27%), T2 (19%), ≥T3 (10%) | Frozen tissues  FFPE | qPCR  IHC | 30% at mRNA level  75% at protein level | 0% at mRNA level  14% at protein level | Overexpression associated with higher grade, invasion and shorter RFS | [[209](#_ENREF_209)] |
| **GPI-specific phospholipase D+H-ras** | Bladder cancer (n=2) | Tumour cell lines | qPCR  Southern blotting  EIA | 100% | Not specified | Co-expression with H-ras oncogene associated with increased malignancy | [[212](#_ENREF_212)] |
| **CD109** | Normal bladder epithelium (n=7)  Urothelial bladder cancer (n=156)  - *Grade:* G1 (19%), G2 (60%), G3 (21%)  - *Stage:* pTa (60%), pT1 (15%), pT2 (8%), pT3 (12%), pT4 (5%) | FFPE | IHC | 70% | 0% | Expression associated with lower grade and stage and better cancer-specific survival | [[213](#_ENREF_213)] |
| **Prostasin (PRSS8)** | Normal bladder epithelium (n=36)  Urothelial bladder cancer (n=37)  - *Grade:* G1 (22%), G2 (46%), G3 (32%) | FFPE  Tumour cell lines | IHC  Western blotting  qPCR | G1 (63%)  G2 (35%)  G3 (17%) | 92% | Loss of expression associated with higher grade | [[216](#_ENREF_216)] |
| **Glypican-3** | Squamous cell carcinomas (n=107)  Invasive urothelial carcinomas (n=49) | Tissues | IHC | 20%  12% | 94% | NS for diagnosis - tissues | [[217](#_ENREF_217)] |
|  | Urothelial bladder cancer (n=384) | FFPE | IHC | 6% | Not specified | NS for stage, grade, invasion, concomitant Tis, soft tissue surgical margins, RFS or cancer specific mortality | [[221](#_ENREF_221)] |
| EIA: enzyme immunoassay; FFPE: formalin-fixed paraffin-embedded tissue; GPI: glycosylphosphatidylinositol; HPLC: high-performance liquid chromatography; HYAL1: hyaluronidase 1; IF: immunofluorescence; IHC: immunohistochemistry; MIA: matrigel invasion assay; MIBC: muscle-invasive bladder cancer; mRNA: messenger ribonucleic acid; NMIBC: non-muscle-invasive bladder cancer; NS: not statistically significant; PCR: polymerase chain reaction; qPCR: quantitative real-time polymerase chain reaction; RCA: red cell adherence test; RFS: recurrence-free survival; RHAMM: receptor for hyaluronan-mediated motility; RMA: radial migration assay; RT-PCR: reverse-transcription polymerase chain reaction; TEM: transendothelial migration assay. | | | | | | | |
